# ZNF714 Supports Pro-Oncogenic Features in Lung Cancer Cells

**DOI:** 10.3390/ijms242115530

**Published:** 2023-10-24

**Authors:** Urszula Oleksiewicz, Marta Machnik, Joanna Sobocińska, Sara Molenda, Anna Olechnowicz, Anna Florczak, Mikołaj Smolibowski, Mariusz Kaczmarek

**Affiliations:** 1 Department of Cancer Immunology, Chair of Medical Biotechnology, Poznan University of Medical Sciences, 8 Rokietnicka Street, 60-806 Poznan, Poland; 2 Department of Diagnostics and Cancer Immunology, Greater Poland Cancer Center, Garbary 15, 61-866 Poznan, Poland; 3 Doctoral School, Poznan University of Medical Sciences, 60-812 Poznan, Poland; 4 Department of Histology and Embryology, Poznan University of Medical Sciences, Swiecickiego 6 Street, 60-781 Poznan, Poland

**Keywords:** ZNF714, oncogenes, transcriptomic profiling

## Abstract

Despite the ongoing progress in diagnosis and treatments, cancer remains a threat to more than one-third of the human population. The emerging data indicate that many Krüppel-associated box zinc finger proteins (KRAB-ZNF) belonging to a large gene family may be involved in carcinogenesis. Our previous study identified Zinc Finger Protein 714 (ZNF714), a KRAB-ZNF gene of unknown function, as being commonly overexpressed in many tumors, pointing to its hypothetical oncogenic role. Here, we harnessed The Cancer Genome Atlas (TCGA)-centered databases and performed functional studies with transcriptomic and methylomic profiling to explore ZNF714 function in cancer. Our pan-cancer analyses confirmed frequent ZNF714 overexpression in multiple tumors, possibly due to regional amplification, promoter hypomethylation, and Nuclear Transcription Factor Y Subunit Beta (NFYB) signaling. We also showed that ZNF714 expression correlates with tumor immunosuppressive features. The in vitro studies indicated that ZNF714 expression positively associates with proliferation, migration, and invasion. The transcriptomic analysis of ZNF714 knocked-down cells demonstrated deregulation of cell adhesion, migration, proliferation, apoptosis, and differentiation. Importantly, we provided evidence that ZNF714 negatively regulates the expression of several known TSGs indirectly via promoter methylation. However, as ZNF714 did not show nuclear localization in our research model, the regulatory mechanisms exerted by ZNF714 require further investigation. In conclusion, our results reveal, for the first time, that ZNF714 may support pro-oncogenic features in lung cancer cells.

## 1. Introduction

Despite enormous research efforts and substantial progress in diagnosis and treatments, cancer remains a threat to more than one-third of the human population and the second leading cause of death [1]. Carcinogenesis is a complex process driven by the aberrant functioning of the genes altered via mutations, chromosomal variations, and epigenetic dysregulation [2,3]. These alterations lead to a cancerous phenotype characterized by sustained proliferation, immortality, resistance to apoptosis, metastasis, altered metabolism, immune response evasion, phenotypic plasticity, and other features [3]. Cancer is an etiologically heterogeneous disease in which a plethora of molecular aberrations within different signaling networks may lead to similar pathogenic manifestations. Tumor heterogeneity is also related to its broad plasticity allowing for the acquisition of various phenotypes, from stem-like to differentiated cells. Upon various insults (e.g., hypoxia, nutrient deprivation, anti-cancer therapy) imposing a selective pressure, cancer tissue may undergo a clonal evolution resulting in an adaptation and/or escape from adverse conditions [4,5]. A comprehensive characterization of the molecular events driving carcinogenesis is urgently needed to improve current diagnostics, treatment options, and patient monitoring. 

Krüppel-associated box zinc finger proteins (KRAB-ZNFs) belong to a gene family in which some of the members have a documented role in various cancers [6]. KRAB-ZNFs constitute a large group of homologous genes (>380) with multiple splicing variants and pseudogenes [7,8]. A classical KRAB-ZNF protein contains an array of zinc finger motifs recognizing a specific DNA sequence and a KRAB domain binding KRAB-Associated Protein 1 (KAP1). KAP1 serves as a scaffold for a protein complex that induces epigenetic repression of affected loci. The complex consists of histone deacetylase (Chromodomain Helicase DNA Binding Protein 3 (CHD3—a subunit of Nucleosome Remodeling and Deacetylase: NuRD complex)), H3K9 methyltransferase SET Domain Bifurcated 1 (SETDB1), and Heterochromatin Protein 1 (HP1), which altogether trigger chromatin compaction [9,10]. KRAB-ZNFs also have various non-canonical functions dependent on interaction with other proteins [6,11]. Due to their homology, little is known about the exact molecular functions of most KRAB-ZNF genes. Nevertheless, an increasing amount of evidence, based mainly on the single-gene analysis, indicates that various KRAB-ZNF factors are implicated in carcinogenesis, functioning as oncogenes or tumor suppressor genes (TSGs) [6,11,12]. In our previous studies [13], we explored a cancer-related signature of KRAB-ZNF genes to detect those KRAB-ZNFs that may be commonly overexpressed in various tumor types. To this end, we utilized The Cancer Genome Atlas (TCGA) transcriptomic datasets and showed that the expression of multiple KRAB-ZNFs is deregulated in many tumor types. This analysis identified 16 frequently overexpressed KRAB-ZNFs, including Zinc Finger Protein 714 (ZNF714) [13].

ZNF714 is a gene with an unknown role in cancer and sparse literature reports about its molecular function. According to our knowledge, there is no report characterizing ZNF714’s role as a stand-alone gene, but rather it appears in various omic datasets linked to specific biological phenomena. We selected ZNF714 for further study due to our previous observations that ZNF714 becomes markedly overexpressed upon dedifferentiation of dermal fibroblasts to induced pluripotent stem cells [14]. Additional evidence of the ZNF714 linkage with stemness comes from the study published by Chung and colleagues [15]. In their analysis, an increased expression of ZNF714 was found in the spheroids formed by the HCT116 colon cancer cell line, together with other genes associated with a cancer stem cell signature. As stemness is an important aspect of tumor biology supporting metastatic events and therapy resistance [16], we speculated that ZNF714 association with stem cell phenotype may also be related to carcinogenesis.

In the current study, we aimed to test the hypothesis that ZNF714 plays an oncogenic role in tumor biology. First, we screened various online tools utilizing TCGA data to explore the status of ZNF714 in a pan-cancer setting. These analyses, built upon our previous study [13], confirmed that ZNF714 mRNA levels are higher in many tumor types compared to normal samples. The data indicated that ZNF714 overexpression is associated with promoter hypomethylation, regional amplification, p53-mutated status, and, possibly, co-regulation with neighboring genes via Nuclear Transcription Factor Y Subunit Beta (NFYB). Moreover, the cancers with high ZNF714 levels exhibited low interactions with the immune system. The in vitro phenotypic studies in the lung cancer model indicated that ZNF714 may support proliferation, migration, and invasion in a cell-type-dependent manner. Although, surprisingly, we observed that ZNF714 does not translocate to the nucleus, its silencing led to the upregulation of several known TSGs and accompanying hypomethylation of their promoters. Altogether, our data suggested that ZNF714 may have a pro-oncogenic role in our research model.

## 2. Results

### 2.1. Exploration of ZNF714 Expression Status in Relation to Clinicopathological Parameters in Various Tumor Types Based on TCGA Samples

**Figure 1:**
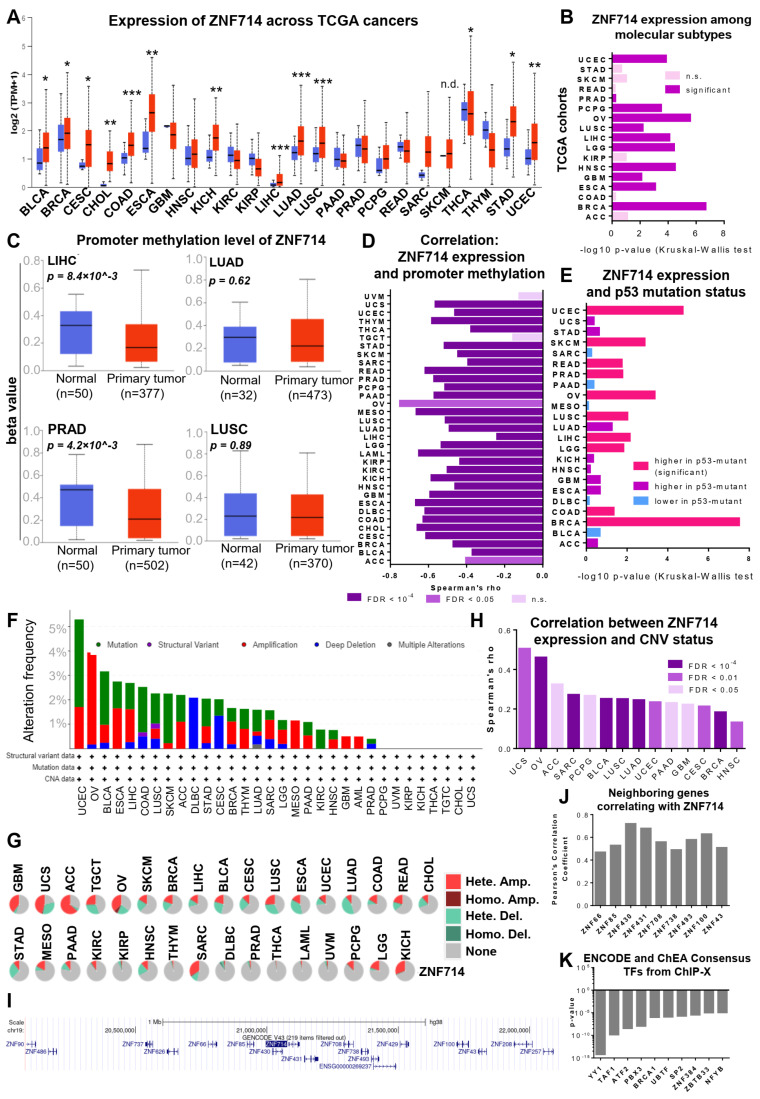
ZNF714 status in TCGA specimens. Boxplot representation of ZNF714 expression (**A**) and promoter methylation (**C**) in normal (blue) and tumor (red) samples across various TCGA cohorts based on the UALCAN database. Student’s *t*-test was used to assess the significance of the difference; * <0.05, ** <10^−5^, *** <10^−10^, n.d. no data. (**B**) ZNF714 expression differs significantly among molecular subtypes in the majority of TCGA cohorts. (**D**) ZNF714 promoter methylation level correlates negatively with ZNF714 expression. (**E**) ZNF714 expression is higher in most of the tumors with mutated p53. (**F**,**G**) Mutational status of ZNF714 throughout various tumor types. (**H**) ZNF714 expression correlates with Copy Number Variation (CNV) status. (**I**) Genome Browser visualization of ZNF714 neighborhood 1 Mb upstream and downstream from TSS and TTS, respectively. The surrounding genes belong to the zinc finger family. (**J**) Pearson’s correlation analysis demonstrates the co-expression of ZNF714 and surrounding genes. (**K**) Transcription factors (TFs) binding to the top 100 genes with similar expression patterns compared to ZNF714 expression identified via EnrichR Encode and ChEA Consensus TFs from ChIP-X analysis. Adrenocortical carcinoma (ACC); Acute Myeloid Leukemia (AML); bladder urothelial carcinoma (BLCA); breast invasive carcinoma (BRCA); cervical squamous cell carcinoma and endocervical adenocarcinoma (CESC); cholangiocarcinoma (CHOL); Colon adenocarcinoma (COAD); lymphoid neoplasm diffuse large B-cell lymphoma (DLBC); esophageal carcinoma (ESCA); glioblastoma multiforme (GBM); head and neck squamous cell carcinoma (HNSC); kidney chromophobe (KICH); kidney renal clear cell carcinoma (KIRC); kidney renal papillary cell carcinoma (KIRP); brain lower-grade glioma (LGG); liver hepatocellular carcinoma (LIHC); lung adenocarcinoma (LUAD); lung squamous cell carcinoma (LUSC); mesothelioma (MESO); ovarian serous cystadenocarcinoma (OV); pancreatic adenocarcinoma (PAAD; prostate adenocarcinoma (PRAD); pheochromocytoma and paraganglioma (PCPG); rectum adenocarcinoma (READ); sarcoma (SARC); skin cutaneous melanoma (SKCM); stomach adenocarcinoma (STAD); testicular germ cell tumor (TGCT); thyroid carcinoma (THCA); thymoma (THYM); uterine corpus endometrial carcinoma (UCEC); uterine carcinosarcoma (UCS); uveal melanoma (UVM).

Our previous analysis of TCGA transcriptomic datasets identified ZNF714 as one of sixteen KRAB-ZNF genes commonly upregulated in many tumor types [13]. We selected ZNF714 for further analysis due to its increased expression during dedifferentiation from mature fibroblasts into induced pluripotent stem cells [14]. This observation indicates the potential involvement of ZNF714 in stemness, which is also a feature important in carcinogenesis [17]. We commenced our in-depth analysis of ZNF714’s involvement in cancer biology by exploration of the online tools utilizing TCGA datasets. First, we monitored ZNF714 mRNA expression in 24 types of normal and tumor tissues. We confirmed that ZNF714 is overexpressed in many tumor types (Figure 1A). The data indicated that in comparison to normal tissues, ZNF714 expression was significantly increased in bladder (BLCA), breast (BRCA), cervix (CESC), colon (COAD), esophagus (ESCA), liver (LIHC), lung (LUAD, LUSC), stomach (STAD), and uterine (UCEC) tumors, as well as in cholangiocarcinoma (CHOL) and kidney chromophobe (KICH). Only thyroid carcinoma (THCA) showed significantly lowered ZNF714 levels (Figure 1A). Second, we observed that ZNF714 expression varies among different molecular subtypes in 10 tumor types, including BRCA, ESCA, glioblastoma (GBM), head and neck cancer (HNSC), low-grade glioblastoma (LGG), LIHC, LUSC, ovarian cancer (OV), pheochromocytoma and paraganglioma (PCPG), and UCEC (Figure 1B, Figure S1A). Third, ZNF714 expression showed a positive correlation with tumor stage (Figure S1B) and grade (Figure S1C) in LIHC and UCEC, which was not the case in any other tumor. We also tested the potential prognostic value of ZNF714 expression. In general, high ZNF714 expression correlated with poorer overall survival in PCPG and mesothelioma (MESO) (Figure S1D) and disease-free survival in uveal melanoma (UVM), kidney renal papillary cell carcinoma (KIRP), and adrenocortical carcinoma (ACC) (Figure S1E). The opposite effect was observed in the case of overall survival in OV patients (Figure S1D).

### 2.2. Search for the Molecular Mechanisms Leading to the Increased Expression of ZNF714 in Cancer

Next, we asked about the potential mechanisms triggering ZNF714 overexpression in cancer. To this end, we looked at the DNA methylation within the ZNF714 promoter. In many tumors, the methylation profile became wider than in normal tissues. The exemplary methylation profiles are presented in Figure 1C. Although in some tumor samples the methylation level increased, the overall median methylation demonstrated a decreasing trend in the majority of tumors (Figure 1C). Of note, CpG methylation within the *ZNF714* promoter inversely correlated with ZNF714 expression. These results were significant in all of the analyzed tumor types, except for testicular germ cell tumors (TGCT) and UVM (Figure 1D). Nevertheless, the comparative analysis of normal and tumor samples indicated that hypomethylation is significant only in LIHC and prostate adenocarcinoma (PRAD) cohorts (Figure 1C). 

In addition, we observed that the ZNF714 mRNA level is higher in the majority of tumors with mutated p53 (Figure 1E). We further looked at the frequency of mutation within the ZNF714 gene using two online tools: cbioportal and GSCA. Both databases demonstrated that point mutations are relatively rare events (Figure 1F and Figure S2A). They occur throughout the whole gene (Figure S2B) and are categorized as passenger mutations by cbioportal algorithms. As far as copy number variations (CNVs) are concerned, both databases showed contradictory results. While cbioportal algorithms called a low frequency of CNVs (up to ~5% in OV, Figure 1F), GSCA CNV events ranged from 1.6% of total amplification and 1% total deletion (in THCA) to 46.4% of total amplification and 32.1% total deletion in uterine carcinosarcoma (UCS) (Figure 1G). These discrepancies may stem from differences in the threshold values utilized by both databases to call a CNV event. Cbioportal seems more restrictive, focusing mainly on deep amplifications and deletions. Notably, CNV status positively correlated with ZNF714 expression in 14 out of 33 tumor types analyzed via the GSCA portal (Figure 1H). 

These observations prompted us to test whether the surrounding genes may be expressed analogously to ZNF714. We selected a genomic area 1 MB upstream from the transcription start site (TSS) and 1 MB downstream from the transcription termination site (TTS) of ZNF714. The fragment contained 15 zinc finger genes, all of which harbored a KRAB domain (except for ZNF493) (Figure 1I). Using GEPIA2 Similar Genes Detection mode, we downloaded the top 100 genes correlating with ZNF714 expression in all TCGA tumor types. A block of nine ZNF714-surrounding zinc finger genes showed a marked co-expression signature with ZNF714 (from ZNF66 to ZNF43) (Figure 1J). Frequent CNV of this locus may be one of the explanations for the co-expression phenomenon. However, these genes may also be co-regulated by a common transcription factor (TF) and/or reside within the same topologically associated domain. To explore the first possibility, we uploaded the above-mentioned list of the top 100 genes (+ZNF714) to the EnrichR website to identify TFs targeting those genes. We distinguished Ying-Yang 1 (YY1), TATA-Box Binding Protein Associated Factor 1 (TAF1), Activating Transcription Factor 2 (ATF2), Pre-B-Cell Leukemia Homeobox 3 (PBX3), and BRCA1 DNA repair associated (BRCA1) as the top 5 consensus TFs from various ChIP experiments from ENCODE and ChEA (Figure 1K and Figure S2C). The analysis detected NFYB (Figure S2C) as the only TF targeting most of the co-regulated neighboring genes (ZNF100, ZNF738, ZNF708, ZNF431, ZNF714, ZNF430).

### 2.3. High ZNF714 Expression Associates with Low Immune Activity within the Tumor

We further inspected the correlation between ZNF714 expression and various aspects related to the interactions between the tumor and the immune system. The analyses performed via the TISIDB portal showed that, in general, ZNF714 expression is associated with low activity of the immune system across most of the analyzed tumors (except for LIHC and THCA) (Figure S3). ZNF714 mRNA level demonstrated a significant correlation with immune subtypes in 8 out 33 TCGA tumor types (Figure S3A). In the majority of cases, ZNF714 expression was low in immune subtypes classified [18] as inflammatory (C3), while high expression was evident for lymphocyte depleted (C4) subtypes (Figure S3A). The exception to this rule was LIHC with a high ZNF714 level in the wound-healing subtype (C1) and low ZNF714 in the lymphocyte-depleted subtype. These observations were further confirmed with the tumor-infiltrating lymphocyte (TIL) signatures, most of which showed a negative correlation with ZNF714 expression in the majority of tumor types tested (Figure S3B). Only active CD4+, effector memory T (Tem CD4+), type 2 T helper (Th2), and memory B lymphocytes positively correlated with ZNF714 level across various tumor types. This signature indicates an association between ZNF714 level and humoral response within tumor tissue. 

Furthermore, the pan-cancer data indicated a positive association between ZNF714 mRNA level and the expression of several immunoinhibitors (Figure S3C). This included CD274 (the gene coding for Programmed Death Ligand 1: PD-L1), Vascular Endothelial Growth Factor Receptor 2 (KDR), Transforming Growth Factor Beta Receptor 1 (TGFBR1), and V-Set Domain Containing T Cell Activation Inhibitor 1 (VCTN1). Additionally, two immunostimulators were associated with ZNF714 expression in a pan-cancer manner (Figure S3D), namely, Tumor Necrosis Factor Superfamily Member 15 (TNFSF15) and UL16 Binding Protein, a ligand activating NK and T cells (ULBP1). In contrast, the correlation between ZNF714 expression and MHC genes, chemokines, and chemokine receptors revealed a tumor-specific pattern (Figure S3E–G). An apparent negative correlation with these genes was observed in CHOL, KICH, and TGCT (Figure S3E–G). As far as a positive association is concerned, MHC genes correlated with ZNF714 mainly in THCA (Figure S3E), with chemokines in CESC, ESCA, LIHC, and THCA (Figure S3F), and with chemokine receptors in ESCA, LIHC, THCA, and UCS (Figure S3G).

### 2.4. ZNF714 Knockdown Decreases the Proliferative Potential of Cancer Cells

**Figure 2:**
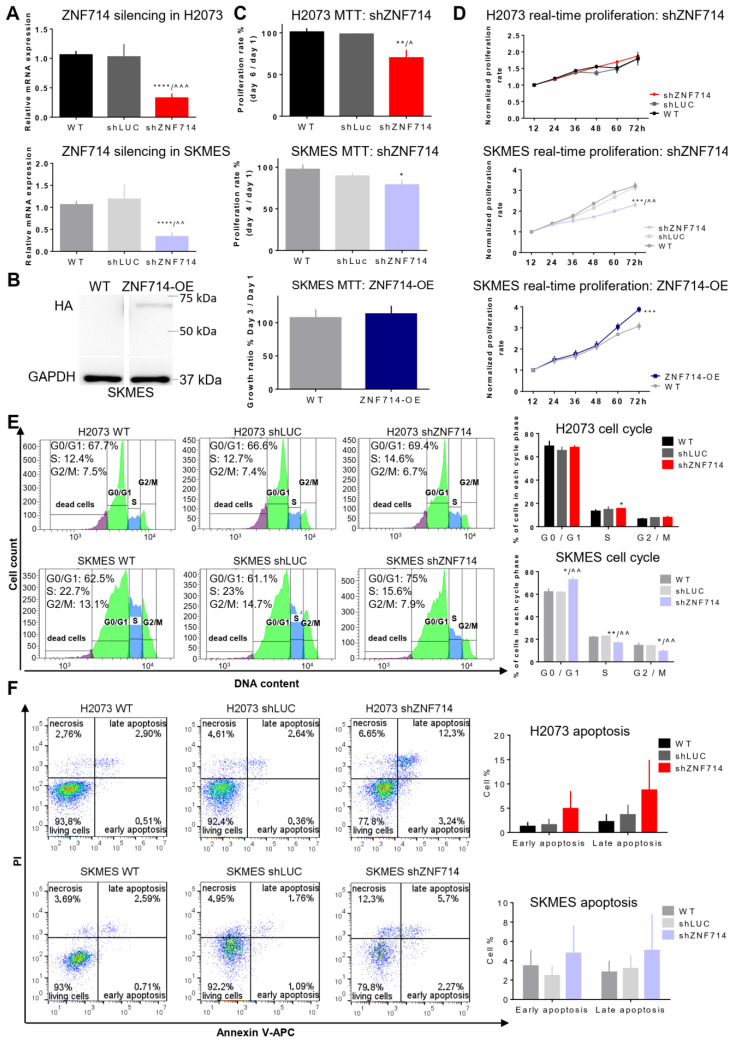
ZNF714 knockdown decreases the proliferative potential of cancer cells. (**A**) The level of ZNF714 gene expression after RNAi-mediated knockdown in H2073 and SKMES cell lines relative to ESD control. (**B**) Forced overexpression of HA-tagged ZNF714 in the SKMES cell line was confirmed by Western blot analysis relative to GAPDH internal control. Cell proliferation after ZNF714 knockdown or overexpression was assessed by MTT colorimetric test (**C**) and real-time proliferation assay on an IncuCyte Instrument (**D**). (**E**,**F**) Cell cycle analysis and apoptosis were determined by flow cytometry. The pictures show representative images for a selected biological replicate. The bar graphs show statistics for 3–5 biological replicates. Statistical analysis was performed with an unpaired *t*-test. *p*-value symbols: * vs. WT; ^ vs. shLUC; *^/^^ *p* < 0.05; **^/^^^ *p* < 0.01; ***^/^^^^ *p* < 0.001, **** *p* < 0.0001. Abbreviations: WT—wild type, unmodified cells; shLUC—cells carrying control shRNA sequence; shZNF714—cells carrying shRNA sequence complementary to ZNF714; OE—overexpression; PI—propidium iodide; HA—hemagglutinin tag.

The data presented above indicated that ZNF714 may play a role in the biology of various tumors. Thus, to further investigate the function of ZNF714 in cancer, we performed a series of gold-standard phenotypic in vitro assays aiming to explore the ZNF714 effect on proliferation, apoptosis, cell cycle, migration, and invasion. We selected the lung cancer model as the most common malignancy with the highest frequency of death in both sexes [19]. Moreover, ZNF714 expression proved to be significantly upregulated in both major histopathological subtypes of lung cancer, namely LUAD and LUSC (Figure 1A) [13]. We used a lentiviral approach to knock down ZNF714 expression with the shRNA molecules (shZNF714) in H2073 (LUAD) and SKMES (LUSC) cell lines (Figure 2A). We also attempted to overexpress ZNF714, but this was possible only in the SKMES cell line (ZNF714-OE) (Figure 2B). Interestingly, the cells with knocked-down expression of ZNF714 exhibited altered morphology, which was most apparent shortly after trypsinization. While the control cells spread into an elongated (SKMES) or cubic (H2073) shape 24 h after passaging, many shZNF714 cells still retained rounded shape suggesting hindered adhesion and spreading (Figure S4). 

In the metabolic MTT proliferation assays, we observed a reduced proliferation rate in both shZNF714 cell lines (Figure 2C). However, no opposite effect was noticed upon ZNF714 overexpression in the SKMES cell line. We also performed a real-time proliferation test based on cell confluency (Figure 2D). Here, the H2073 cell line showed no difference in the growth of all variants. However, SKMES shZNF714 grew at a slower rate, whereas ZNF714-OE had increased proliferation potential (Figure 2D). We also silenced ZNF714 expression in two breast cancer cell lines, namely MDA-MB-468 (basal breast cancer) and MCF7 (luminal A) [20] (Figure S5A). These cells were also subjected to MTT and confluency-based proliferation assays. The results demonstrated a decreased growth rate of MDA-MB-468 shZNF714 compared to controls, but only in the MTT test (Figure S5B,C). These data indicate that ZNF714 may positively influence the proliferation capacity of the cancer cells; however, the effect depends on the type of assay and cell lines used.

Next, we wanted to investigate potential mechanisms supporting lowered proliferative potential in shZNF714 cell lines by focusing on cell cycle and apoptosis. The cell cycle analysis showed limited differences between H2073 cell line variants as shZNF714 showed a minor increase in the S phase population compared to unmodified wild-type (WT) cells (Figure 2E). However, SKMES shZNF714 cells showed a significant increase in G0/G1 phase and a simultaneous decrease in S and G2/M phases, indicating a cell cycle arrest evoked by ZNF714 silencing (Figure 2E). An opposite situation was observed in the annexin V apoptosis assay. Here, H2037 and SKMES shZNF714 cells demonstrated a trend of higher population within early and late apoptosis compared to both controls (Figure 2F). Both sets of breast cancer cell lines showed no difference in the cell cycle and apoptosis assays. Our observations suggest that the mechanisms driving reduced growth rate in cancer cells upon ZNF714 knockdown are cell-type dependent.

### 2.5. ZNF714 Positively Affects Cell Migration and Invasiveness

**Figure 3:**
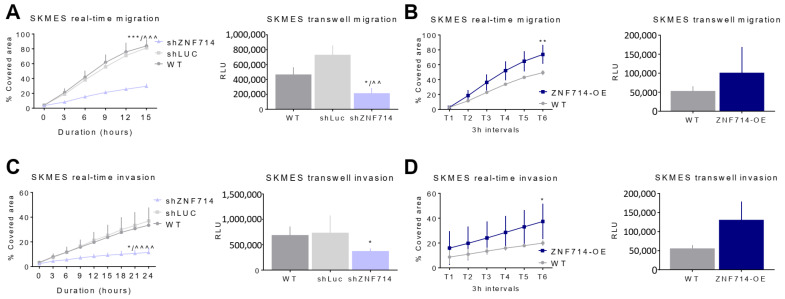
ZNF714 positively affects cell migration and invasiveness. Migration and invasion level in SKMES cell line with shZNF714 (**A**,**C**) and ZNF714-OE (**B**,**D**). The cells were subject to real-time wound healing assays on IncuCyte (**A**,**B**) and a Boyden chamber transwell assay with chemoattractant (**C**,**D**). Statistical analysis was performed with an unpaired t-test. *p*-value symbols: * vs. WT; ^ vs. shLUC; * *p* < 0.05; **/^^ *p* < 0.01; ***/^^^ *p* < 0.001, ^^^^ *p* < 0.0001.

To investigate ZNF714’s influence on cell migration and invasion, we applied wound healing and Boyden chamber assays, in which Matrigel coating served as a medium for invasion. Only the SKMES cell line migrated at a sufficient rate to allow the monitoring of the changes in migration and invasion velocity upon altered ZNF714 expression. The remaining cell lines (H2073, MDA-MB-468, and MCF7) demonstrated very low basal migration and invasion; hence, they did not provide any interpretable data. Our analyses revealed that ZNF714 silencing reduces, while ZNF714 overexpression increases, cell migration and invasion in both assay types (Figure 3A–D). Although this effect did not reach statistical significance in ZNF714-OE cells in the transwell assay (Figure 3D), the cells did show a trend of higher migration and invasion. These observations suggest that ZNF714 may support the migratory and invasive potential of cancer cells. In summary, the results of in vitro assays point toward the oncogenic function of ZNF714 in our research model. 

### 2.6. Transcriptomic Profile of Lung Cancer Cells with Silenced Expression of ZNF714

**Figure 4:**
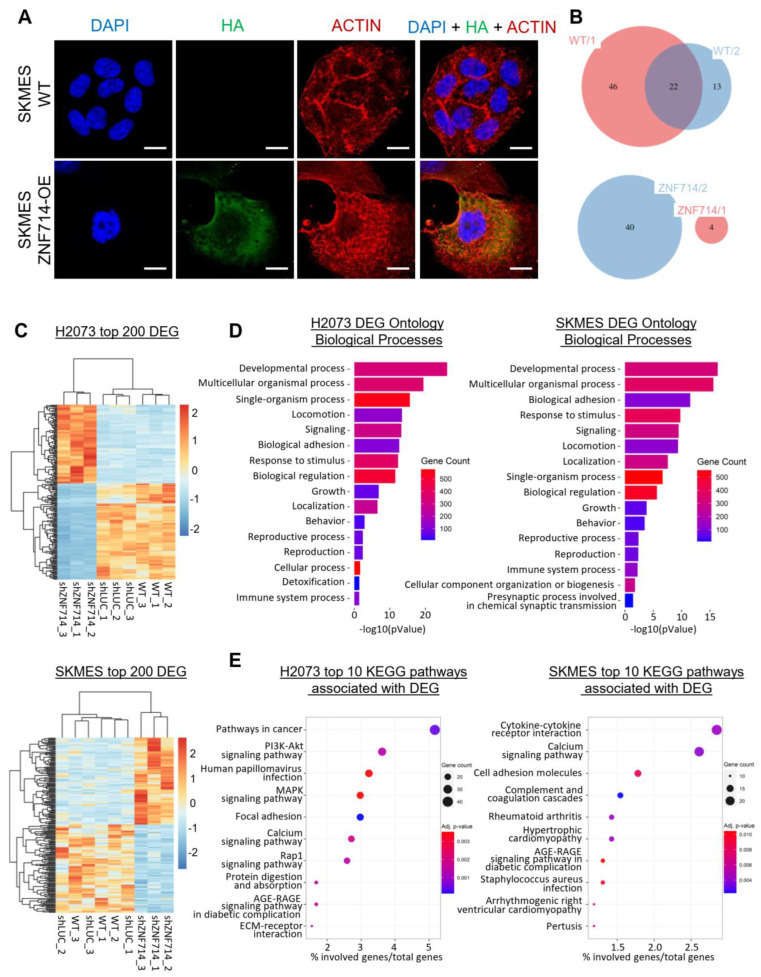
Transcriptomic profile of lung cancer cells with silenced expression of ZNF714. (**A**) ZNF714 localization in SKMES WT and ZNF714-OE cells. Confocal microscopy pictures of anti-HA antibody connected with Alexa Fluor^®^488-conjugated anti-rabbit secondary antibody and DyLight 650-labeled anti-actin antibody. Nuclei were stained with DAPI. Olympus Scanning Confocal Microscope FV1000, 60×. The scale bar represents 10 μm. (**B**) The number of identified peaks in SKMES WT and ZNF714-OE within two replicates in ChIP-seq analysis. (**C**) Z-score normalized heatmaps and supervised clustering representing top 200 (sorted by *p*-value) differentially expressed genes (DEGs) between control cells (WT and shLUC) and shZNF714 cells for H2073 (**top**) and SKMES (**bottom**). The cutoff threshold was fc ≥ 2 or ≤−2, *p* < 0.05. (**D**) Barplots showing enrichment of biological processes (BP1) in all DEGs identified for H2073 (**left**) and SKMES (**right**). The color scale represents the number of DEGs involved in each process. (**E**) Bubble plots representing the top 10 KEGG pathways (sorted by *p*-value) associated with DEGs. *X*-axis represents the ratio of genes involved in each pathway relative to all DEGs. The color scale indicates the *p*-value, and the bubble size represents the number of genes involved in each pathway.

ZNF714 is a KRAB-ZNF gene containing an array of 14 zinc fingers (https://www.uniprot.org/uniprotkb/Q96N38/entry, accessed on 25 July 2023), motifs that are known to attach to specific DNA sequences (https://www.ebi.ac.uk/interpro/entry/pfam/PF00096/#PUB00020583 accessed on 25 July 2023) [21]. Moreover, the KRAB domain mediates epigenetic repression via the KAP1/SETDB1/NuRD/HP1 protein complex [6]. Therefore, we aimed to identify the genomic targets bound and directly regulated by ZNF714 using a ChIP-seq approach. First, we wanted to confirm the nuclear localization of ZNF714. To our dismay, however, the immunocytochemical analysis revealed that ZNF714 resides in the cytoplasm of SKMES ZNF714-OE cell line, whereas the nucleus is dim for anti-HA tag staining (Figure 4A). Of note, the control HA-tagged ZFP57, another KRAB-ZNF protein, showed expected nuclear staining, indicating that the HA tag does not interfere with the transport to the nucleus (Figure S6). Further ChIP-seq analysis on the ZNF714-OE cell line compared to the unmodified WT confirmed the lack of interaction between ZNF714 and nuclear DNA (Figure 4B). The number of identified peaks in ZNF714 was below the WT background, and the peaks differed in both replicates. For this reason, we did not consider these as reliable results.

We further moved to RNA-seq transcriptomic profiling of shZNF714 variants in H2073 and SKMES. We identified over 1000 significantly differentially expressed genes (DEGs) for both cell lines: H2073 (1113 compared to WT and 1314 compared to shLUC) and SKMES (1441 compared to WT and 1048 compared to shLUC). DEGs were integrated to narrow down the list only to those genes that become deregulated in shZNF714 in comparison to both controls. The integration resulted in the sets of 807 DEGs in H2073 and 864 DEGs in SKMES that are commonly up- and downregulated compared to WT and shLUC (Figure 4C). We observed several deregulated KRAB-ZNFs among DEGs, both with increased and decreased expression after ZNF714 knockdown (Table S1). The Gene Ontology (GO) of Biological Processes (BP) in both cell lines pinpointed alterations within developmental processes, locomotion, biological adhesion, response to stimulus, growth, immune system processes, and other (Figure 4D). Additionally, Kyoto Encyclopedia of Genes and Genomes (KEGG) enrichment analysis in both cell lines indicated pathways perturbed in shZNF714 cells, including cell adhesion, Extracellular Matrix (ECM)–receptor interaction, Tumor Necrosis Factor (TNF) signaling, various metabolic pathways [e.g., calcium signaling, Advanced Glycation Endproducts (AGE)-Receptor for AGE (RAGE) signaling pathway in diabetic complications], and disease-related pathways (e.g., cardiomyopathy, viral infections) (Figure 4E). Moreover, in H2073 shZNF714 we observed overrepresentation of the pathways in cancer, pathways related to small lung cancer, and Mitogen-Activated Protein Kinase (MAPK) and Phosphoinositide 3 Kinase (PI3K)-Serine-Threonine Protein Kinase (Akt) signaling (Figure 4E). In SKMES shZNF714 cells, DEGs were also associated with cytokine signaling, including Interleukin 17 (IL-17) signaling (Figure 4E). Cellular components affected by identified DEGs were similar in both cell lines and the pinpointed membrane, extracellular region, and cell junction (Figure S7). The above findings highlight the involvement of ZNF714 in the processes and pathways that are crucial for cancer development.

### 2.7. ZNF714 Inactivates Tumor Suppressor Genes via DNA Methylation

**Figure 5:**
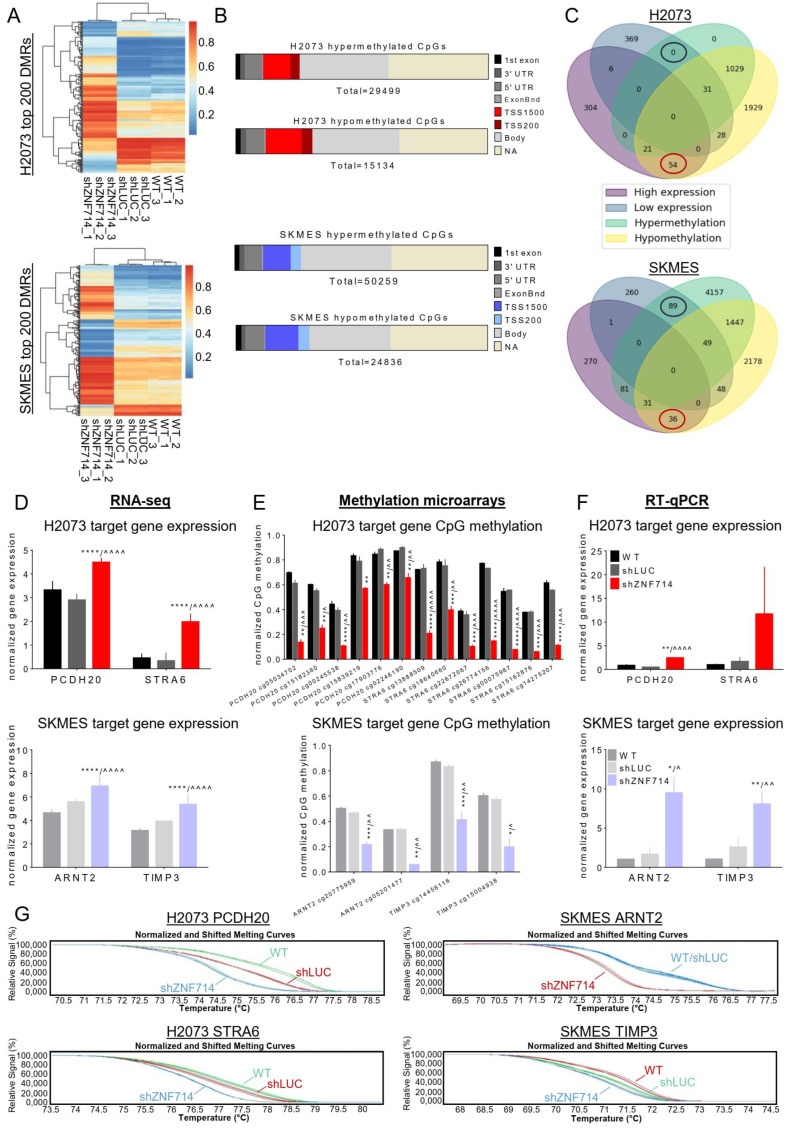
ZNF714 inactivates tumor suppressor genes via DNA methylation. (**A**) Heatmap and hierarchical clustering of top 200 differentially methylated regions (DMRs) (sorted by *p*-value) for H2073 (top panel) and SKMES (bottom panel). The color scale represents delta mean values. (**B**) Bars represent the distribution of hyper- and hypomethylated CpGs across different genome parts. Horizontal slices represent the percentage of CpGs in each region related to all differentially methylated CpGs for each cell line separately. TSS200/TSS1500–distance (in base pairs) from transcription start site. (**C**) Venn diagram integrating DMRs and DEGs in H2073 and SKMES cell lines. Hypo- and hypermethylation refers to cytosines in the TSS gene region. (**D**) Normalized expression of selected ZNF714 target genes in RNAseq analysis. (**E**) Differentially methylated CpGs within the TSS region of selected target genes evaluated by methylation microarray. (**F**) RT-qPCR analysis of selected target gene expression. The experiment was performed in biological and technical triplicates. Statistical analysis was performed with an unpaired *t*-test. */^ *p* ≤ 0.05; **/^^ *p* ≤ 0.01; ***/^^^ *p* ≤ 0.001; ****/^^^^ *p* ≤ 0.0001. * vs. WT, ^ vs. shLUC. (**G**) MS-HRM analysis of DNA methylation within the promoter region of selected target genes. The experiment was performed in biological and technical triplicates. The figure shows representative normalized and shifted melting curves for each gene.

Moreover, we were interested to see whether observed changes in the transcriptomic profile evoked by ZNF714 depletion may be caused by the alterations within the DNA methylome. Thus, H2073 and SKMES cell lines with the knocked-down expression of ZNF714 were subjected to methylation microarray analysis. After integration of all differentially methylated regions (DMRs) in shZNF714 common for the comparisons with WT and shLUC, we identified 45k DMRs in H2073 and 75k DMRs in SKMES (Figure 5A,B). In both cell lines, the majority of changes (both hypo- and hypermethylation) occurred within gene bodies and promoter regions [200–1500 bases upstream of transcription start site (TSS): TSS1500 and 0–200 bases upstream of TSS: TSS200] (Figure 5B). The only visible pattern of altered methylation indicated more frequent hypomethylation within the promoter region in both shZNF714 cell lines (Figure 5B). Next, we searched for CpGs within gene promoter regions with decreased methylation levels in shZNF714 cells compared to both controls. The results were further correlated with RNA-seq results to find target genes that are simultaneously overexpressed and demethylated in shZNF714 cells. We identified 54 genes in H2073 and 36 in SKMES that met our criteria (Figure 5C, Supplemental File S1). Interestingly, several of those genes have documented tumor-suppressive functions, further supporting our in vitro results demonstrating an oncogenic role of ZNF714. 

We focused our attention on four target genes: Protocadherin 20 (*PCDH20*) [22,23] and Signaling Receptor And Transporter Of Retinol (*STRA6*) [24,25,26] for H2073, and Aryl Hydrocarbon Receptor Nuclear Translocator 2 (*ARNT2*) [27,28,29] and Tissue Inhibitor Of Metalloproteinases 3 (*TIMP3*) [30,31,32] for the SKMES cell line. Gene expression and DNA methylation of selected genes were significantly deregulated in shZNF714 cells based on RNA-seq and methylation microarray analysis (Figure 5D,E; Table 1). We validated these results with single-gene assays: RT-qPCR for gene expression and Methyl-Specific High Resolution Melting (MS-HRM) for DNA methylation. In H2073, the expression level of PCDH20 and STRA6 was elevated in shZNF714 cells (Figure 5F) and DNA methylation was decreased (Figure 5G). Similarly, in SKMES, after ZNF714 knockdown, ARNT2 and TIMP3 were overexpressed (Figure 5F), while their promoters became demethylated (Figure 5G). These results confirmed the observed correlation between RNA-seq and methylation microarray and indicated that ZNF714 might be involved in carcinogenesis by negatively regulating TSGs.

### 2.8. Common Dysregulation of Gene Expression and DNA Methylation in Lung Cancer Cell Lines

**Figure 6:**
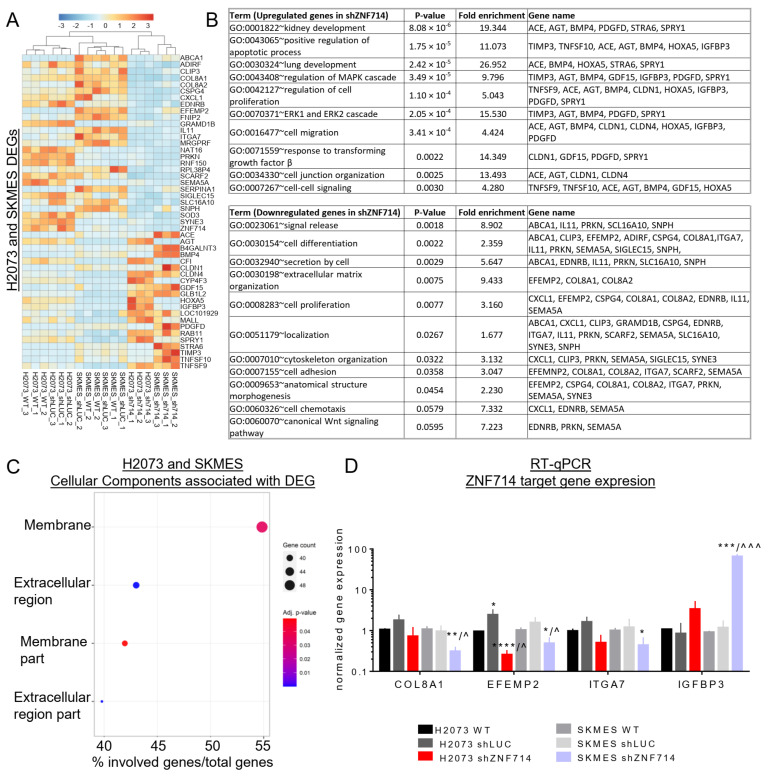
Common deregulation of gene expression and DNA methylation in lung cancer cell lines. (**A**) Z-score normalized heatmap representing all DEGs similarly deregulated in H2073 and SKMES cell lines after ZNF714 knockdown. (**B**) Table showing biological processes (BP ALL) connected to DEGs that are similarly deregulated in H2073 and SKMES cell lines after ZNF714 knockdown. (**C**) Bubble plots representing cellular components (CC1) associated with DEGs. *X*-axis represents the ratio of genes involved in each pathway relative to all DEGs. The color scale indicates the *p*-value and the bubble size represents the number of genes involved in each pathway. (**D**) RT-qPCR analysis of selected ZNF714 target gene expression. Statistical analysis was performed with an unpaired *t*-test. */^ *p* ≤ 0.05; **p ≤ 0.01; ***/^^^ *p* ≤ 0.001; **** *p* ≤ 0.0001. * vs. WT, ^ vs. shLUC.

Furthermore, we asked whether ZNF714 may affect gene expression in both analyzed lung cancer cell lines. Hence, we integrated the expression datasets for H2073 and SKMES, which resulted in a list of 21 commonly upregulated and 26 commonly downregulated genes in shZNF714 cell variants (Figure 6A). The gene ontology analysis identified several biological processes that contained a different combination of the genes in each category (up- and downregulated). The complete list of all identified biological processes is included in Supplemental File S2, whereas the non-overlapping, most interesting ontologies are presented in Figure 6B. In summary, we discovered that the genes upregulated upon ZNF714 silencing in both cell lines are related to kidney and lung development, positive regulation of apoptotic processes, MAPK and ERK1/ERK2 cascade, cell–cell signaling, and cell junction organization. The downregulated genes fall into the category of signal release, secretion, cell differentiation, anatomical structure morphogenesis, extracellular matrix organization, localization, adhesion, chemotaxis, and the canonical Wnt signaling pathway. Both up- and downregulated genes were associated with cell proliferation and motility, the processes we observed to be altered in our in vitro experiments. Moreover, DEGs were overrepresented in the cell membrane and extracellular region (Figure 6C). We further selected a few genes for wet-lab validation with the single-gene qPCR assays. We chose the genes based on the participation in the biological processes that were also altered in our in vitro studies, such as cell proliferation, migration, adhesion, and extracellular matrix organization. We confirmed that Integrin Subunit Alpha 7 (ITGA7), Collagen 8A1 (COL8A1), and EGF Containing Fibulin Extracellular Matrix Protein 2 (EFEMP2) are downregulated, while Insulin Like Growth Factor Binding Protein 3 (IGFBP3) is upregulated in shZNF714 in H2073 and SKMES cell lines (Figure 6D). Altogether, these data show that ZNF714 may affect the expression of various genes participating in many processes important for tumor biology.

## 3. Discussion

KRAB-ZNF factors constitute a large group of homologous genes with poorly characterized molecular functions. Our previous study based on the TCGA transcriptomic profiling identified ZNF714 as one of the 16 KRAB-ZNF genes commonly overexpressed in many tumor types [13]. So far, no literature report elaborates on the biological function of ZNF714 as a stand-alone gene, and only a few omic datasets point towards the association of ZNF714 with various biological processes. As ZNF714 expression was previously shown to increase in induced pluripotent stem cells [14], we hypothesized that due to its linkage with stemness, a feature important for many tumors [3,16], ZNF714 may also be implicated in tumor biology. Thus, we wanted to explore the cancer-related functions of ZNF714 using TCGA-centered online databases, as well as in vitro phenotypic studies and multi-omic analyses in lung cancer cell line models.

The initial analysis confirmed and broadened our previous observations [13]. We found that the ZNF714 mRNA level is significantly higher in 12 out of 24 tumor types compared to normal tissues, whereas lower ZNF714 expression was noted only in THCA (Figure 1A). While this is an important observation implying a potential involvement of ZNF714 in cancer, it will be crucial to validate the transcriptomic data at the protein level. ZNF714 protein has not been systematically studied before, possibly due to the high homology within the KRAB-ZNF gene family and consequent low specificity of available antibodies. We attempted to probe the endogenous ZNF714 protein levels in a panel of lung cancer cell lines; however, we failed to detect the specific ZNF714 band with a commercially available antibody. Moreover, the Human Protein Atlas contains evidence only at the transcript level in the case of ZNF714 (https://www.proteinatlas.org/search/znf714, accessed on 5 September 2023). To date, there is only one article reporting the detection of ZNF714 protein via immunoprecipitation (utilizing an antibody recognizing the zinc finger linker region conserved in many KRAB-ZNFs) coupled with mass spectrometry in the MDA-MB-231 cell line [33]. Future studies should focus on ZNF714 protein expression in a pan-cancer setting.

We also showed that ZNF714 expression was associated with molecular subtypes in many tumor types (10 out of 17) (Figure 1B and Figure S1A), whereas the association with patient prognosis, tumor stage, and grade was rare (Figure S1B–E). Moreover, ZNF714 expression positively correlated with the molecular signatures corresponding to the immunosuppressive phenotype in the majority of tumors (Figure S3). Interestingly, ZNF714 expression showed a positive association with the expression of CD274, which is a gene coding for PD-L1, a checkpoint protein that blocks T cell activation, allowing the tumor to evade the immune system [34]. Indeed, our experimental data revealed that ZNF714 silencing led to the deregulation of immune system processes and cytokine signaling. It would be interesting to test whether the tumors with high ZNF714 may benefit from anti-PD-L1 treatment or whether ZNF714 silencing may restore tumor immunogenicity leading to a decreased tumor expansion. 

Our following analytical workflow concentrated on other aspects related to the potential molecular alterations of ZNF714 in cancer. First, we indicated that *ZNF714* promoter methylation exhibited a significant inverse correlation with its expression (Figure 1D). Although the promoter methylation values become more spread in cancer, we observed an evident trend of hypomethylation in tumors compared to normal specimens (Figure 1C). Of note, several reports suggest that *ZNF714* promoter methylation status is functionally significant [35,36,37,38]. *ZNF714* promoter was classified as a somatic imprinted region undergoing maternal hypermethylation in 43.9% of individuals [35]. Its decreased promoter methylation and accompanying higher mRNA levels in visceral adipose tissues were associated with insulin resistance in an obese subject [36]. The *ZNF714* promoter was also shown to be hypomethylated in monozygotic twins with ovarian cancer compared to their healthy non-twin siblings [37]. It could be speculated that, at least in some tumors, overexpression of ZNF714 may result from its promoter hypomethylation, potentially as a loss-of-imprinting event.

Next, we investigated the frequency of point mutations within the *ZNF714* gene, which appeared to be rare (Figure S1A,B). According to cbioportal algorithms, *ZNF714* mutations may be regarded as passengers. However, a recent multi-modal computational search for driver mutations identified ZNF714 as a putative driver in several cancers, including LUSC. This search was based on the enrichment of cancer-related mutations within CATH domains, the evolutionary-related protein domains with similar structures and functions [39]. The parallel analyses of copy number variation (CNV) events spanning a region with the *ZNF714* gene were contradictory, as they showed both low and high frequency in the cbioportal and GSCA databases, respectively. This contradiction may be related to the differences in the threshold applied by both algorithms to call a CNV. Of note, cbioportal calculations search for deep amplifications and deletions. Nevertheless, amplifications appear more frequently than deletions in both analyses. Moreover, CNV status positively correlates with the ZNF714 mRNA level. ZNF714 is also strongly associated with the expression of its neighboring genes, further implying the overrepresentation of this locus in cancer samples. A study by Pierga and colleagues may partially corroborate this observation. In their analysis, the genomic region harboring *ZNF714* (and other genes, including its closest neighbors: *ZNF85, ZNF430, ZNF431, ZNF708*) was found to undergo copy number gains in estrogen-negative (ER-) breast tumors as compared to ER+ tumors [40]. Nevertheless, the transcriptomic co-regulation of ZNF714 and its neighbors may also be explained by their putative residence within the same topologically associated domain or by an action of a common transcription factor (e.g., NFYB, as detected in our research). In summary, our analysis implies that various mechanisms may contribute to the overexpression of ZNF714, including promoter hypomethylation, local amplifications, and, possibly, NFYB signaling. 

The data generated via TCGA-based databases indicate a potential oncogenic function of ZNF714, and our experimental evidence supports this hypothesis. We chose lung cancer as a research model for the phenotypic and multi-omic analyses because it is the most common tumor among men and women [19]. We selected two lung cancer cell lines from the most prevalent histological subtypes (LUAD and LUSC). We found that ZNF714 silencing leads to decreased proliferation, cell cycle arrest, and augmented apoptosis (Figure 2). Nevertheless, the effect was not uniform among the cell lines and tests, indicating that ZNF714 functioning depends on the molecular background of the cells. Of note, the lung cancer cell lines used in the study were derived from diverse histological subtypes. We also discovered that ZNF714 expression positively correlates with migratory and invasive cell properties (Figure 3). According to the best of our knowledge, this is the first study demonstrating that ZNF714 supports pro-oncogenic features in cancer cell lines. Only one report reveals that increased expression of ZNF714 is associated with the mitotic cell cycle machinery in the normal human dermal fibroblasts undergoing S/G2-M cell cycle phases [41]. 

Transcriptomic RNA-seq profiling of shZNF714 cell lines supports the results obtained in the in vitro assays. Specifically, it demonstrated alterations within such biological processes as growth, locomotion, and adhesion (Figure 4D,E). We showed that ZNF714 silencing interferes with the expression of various growth factors, cytokines, integrins, collagens, and protocadherins. Some upregulated genes were also subject to concomitant promoter hypomethylation, as observed via methylation microarrays. We confirmed the expression and methylation status of a few of the genes with the single-gene assays. Importantly, some the genes upregulated upon ZNF714 knockdown (PCDH20, STRA6, ARNT2, TIMP3) were previously shown to function as tumor suppressors [22,26,27,28,29,42,43,44]. ARNT2 downregulation was observed in several cancers, i.e., hepatocellular carcinoma [27], oral squamous cell carcinoma [29], and lung cancer [28]. In lung and hepatocellular cancer patients, high levels of ARNT2 expression were positively correlated with overall survival (OS), suggesting its potential as an independent prognostic factor for OS. Further phenotypic studies revealed that overexpression of ARNT2 has tumor-suppressive abilities reflected in lower proliferation, migration, and invasion, and higher apoptosis in cancer cell lines and reduced tumor growth in vivo [27,28]. Another factor, PCDH20, was shown to serve as a tumor suppressor in ESCA [44], HNSC [22,43], hepatocellular carcinoma [45,46], and non-small cell lung cancer [23]. In many cases, the downregulation of PCDH20 was linked to its promoter hypermethylation [22,23,44,45]. TSG functions of PCDC20 (inhibition of cell proliferation, migration, invasiveness, epithelial-to-mesenchymal transition, etc.) were shown to be mediated via inhibition of the β-catenin signaling pathway [22,43,44,45]. The *TIMP3* promoter is also frequently subject to hypermethylation [30], whereas in our study, ZNF714 silencing evoked *TIMP3* demethylation. TIMP3 inhibits extracellular matrix proteolytic degradation, thus influencing cell adhesion, migration, proliferation, and apoptosis [47]. Another upregulated and demethylated gene in H2073 cells, STRA6, was reported as an oncogene [24,25], supporting the maintenance of cancer stem cell characteristics [48]. However, it was also shown to contribute to the p53-dependant apoptosis [26] and retinoic acid-induced differentiation pathways [49,50,51]. Thus, the exact role of STRA6 in cancer and stem cells remains to be fully elucidated. Although ZNF714 may affect the promoter methylation and expression of various genes, it needs to be emphasized that ZNF714 does not impose a direct transcriptional regulation over these genes, unlike other KRAB-ZNF repressors. Other yet-unidentified signaling pathways regulated by ZNF714 are likely to be engaged to confer these epigenetic and transcriptomic alterations. Of note, ZNF714 presented a cytoplasmic localization without translocation into the nucleus (Figure 4A), at least in our research model. Nevertheless, previous ChIP-exo experiments identified genomic regions bound by ZNF714 [52], proving that ZNF714 may localize to the nucleus and interact with DNA in certain biological contexts.

In the last steps of our analyses, we identified a relatively small group of genes whose expression becomes commonly deregulated upon ZNF714 knockdown in both lung cancer cell lines (Figure 6A). Their ontology fell into the cancer-related categories, such as regulation of apoptotic processes, proliferation, migration, adhesion, ECM organization, and others (Figure 6B). Interestingly, we identified an upregulated gene set responsible for lung development, which suggests that ZNF714 silencing may reduce cell potency. In our validation assays, we tested three downregulated genes with known oncogenic properties (Figure 6D), namely EFEMP2 [53,54], ITGA7 [55,56,57], and COL8A1 [58,59,60]. These genes participate in cell adhesion, extracellular matrix organization, cell proliferation, and anatomical structure morphogenesis (Figure 6B). Decreased expression of EFEMP2, ITGA7, and COL8A1 may likely affect these processes. The alterations within these processes may at least partially explain changes in the morphology, longer cell recovery after trypsinization, and reduced migration and invasiveness of shZNF714 cells. We also assayed IGFBP3, an upregulated gene with a documented TSG function (Figure 6D) [61,62,63]. Interestingly, previous reports pointed toward the involvement of ZNF714 in diabetes [64] and insulin resistance [36]. High near-birth ZNF714 expression was incorporated into the predictive model identifying patients at risk of type 1 diabetes [64]. As mentioned previously, high ZNF714 expression accompanied by promoter hypomethylation was detected in the visceral adipose tissues of obese, insulin-resistant patients [36]. It remains to be tested whether ZNF714 may interfere with insulin metabolism via the downregulation of IGFBP3 expression in other research models.

## 4. Materials and Methods

### 4.1. Databases Used for the Analysis of Pan-Cancer Omics Data Related to ZNF714

The UALCAN database (https://ualcan.path.uab.edu/, accessed on 17 May 2023) [65,66] was used to assess the differential expression of ZNF714 in a pan-cancer setting. We compared the expression between normal and cancer samples and wild-type and mutated p53 tumors. Moreover, we explored the changes in DNA methylation within the ZNF714 promoter. 

The TISIDB database (http://cis.hku.hk/TISIDB/index.php, accessed on 22 May 2023) [67] was used to probe the differences in ZNF714 expression among various molecular and immune subtypes, and tumor stages and grades. In addition, we analyzed the relation between ZNF714 expression and various features indicative of tumor–immune system interactions (i.e., presence of immune cells, expression of chemokines, chemokine receptors, MHC molecules, immunostimulators, and immunoinhibitors). 

The association between ZNF714 expression level and patient prognosis was tested within the GEPIA2 portal (http://gepia2.cancer-pku.cn/#index, accessed on 14 June 2023) [68]. Median ZNF714 expression served as a cut-off point for separating patients into high and low expression groups in the log-rank test and hazard ratio estimation. GEPIA2’s Similar Genes Detection mode was used to detect the top 100 genes with the most similar expression pattern across all tumor types in the database. These were further uploaded into the EnrichR portal (https://maayanlab.cloud/Enrichr/, accessed on 14 June 2023) [69,70,71] to seek possible common transcription factors regulating their expression. We utilized the hg38 genome from the Genome Browser (https://genome.ucsc.edu/, accessed on 13 June 2023) [72] for the visualization of the ZNF714 neighborhood within a +/− 1 Mb distance from TSS and TTS, respectively.

Mutation status of ZNF714 was evaluated with the aid of cbioportal (https://www.cbioportal.org/, accessed on 18 May 2023) [73,74] and GSCA (http://bioinfo.life.hust.edu.cn/GSCA/, accessed on 7 June 2023) [75,76] algorithms. The latter pinpointed the possible causes of altered ZNF714 expression in tumors by correlating it with CpG methylation and CNV status. Spearman’s correlation results were obtained based on the CpG site whose methylation most negatively correlated with the expression.

### 4.2. Cell Line Model Preparation for ZNF714 Gene Expression Knockdown and Overexpression

For the generation of the in vitro research models, we used American Type Culture Collection (ATCC, USA) cell lines established from lung adenocarcinoma: H2073 (#CRL-5918); lung squamous cell carcinoma: SKMES (#HTB-58); triple-negative breast cancer: MDA-MB-468 (#HTB-132); and luminal A breast cancer: MCF7 (#HTB-22). Lung cancer cell lines were obtained from Dr. Triantafillos Liloglou from the University of Liverpool (UK). HEK-293T (#CRL-3216, ATCC) was used for the preparation of lentiviral particles. Lung cancer cells received DMEM F-12 medium (Biowest, Nuaillé, France), while breast cancer cells and HEK-293T cells were cultured in DMEM High Glucose medium (Biowest, France). The media were supplemented with 10% FBS (EURx, Gdansk, Poland) and 1% penicillin-streptomycin (Biowest, France). The cells were cultured in a humidified incubator at 37 °C in 5% CO_2_. Trypsin (Biowest, France) at the optimized concentration was used to detach the cells during passaging.

Lung and breast cell lines were transduced with lentiviral vectors carrying shRNA sequences complementary to ZNF714 mRNA or to luciferase (5′-CAGCGATGACGAAATTCTTAG-3’) as a control. We used a mix of two different shZNF714 sequences (5′-ACACCTACATCAACATAAAAG-3’; 5′-CAAATGTGGTTGAGTGTAAGG-3’) to obtain the most efficient level of gene knockdown (shZNF714). Lentiviral particles were prepared via a 2nd generation system in HEK-293T cells, using psPAX2 and pMD2.G packaging plasmid and pLV-THEM-GP transfer vector. pLV-THEM-GP was constructed in our laboratory by cloning an additional EcoRI restriction site to pLVTHM (Addgene plasmid # 12247), which was a gift from Prof. Didier Trono [77]. The level of gene expression after knockdown was measured by RT-qPCR relative to ESD (lung) or ACTB (breast) internal control. The same lentiviral packaging system was used to obtain ZNF714 and ZFP57 overexpression (ZNF714-OE and ZFP57-OE, respectively) in the SKMES cell line. HA-tagged KRAB-ZNFs’ coding sequences were delivered on pEXPpSIN-TRE-GW-ZNF714-3xHA and pEXPpSIN-TRE-GW-ZFP57-3xHA transfer plasmids generously provided by Prof. Didier Trono [52]. We used 2 µg/mL doxycycline to induce ZNF714 and ZFP57 overexpression. HA-tagged ZFP57 overexpressing cells were used as a positive control in our immunocytochemistry studies.

### 4.3. Western Blot

The cells were washed with DPBS (Biowest, France) and lysed in RIPA buffer with Protease Inhibitors (Sigma Aldrich, St. Louis, MI, USA) for 30 min on ice. Cell lysates were centrifuged (15,000 rpm/4 °C/15 min), and the supernatant was collected. Protein concentration was determined via BCA reaction with a Pierce™ BCA Protein Assay Kit (Thermo Fisher Scientific, Waltham, Massachusetts, USA), according to the manufacturer’s protocol. Next, 50 μg of protein was mixed with Laemmli Sample Buffer (BioRad, Hercules, CA, USA) and denatured (98 °C/5 min). Electrophoresis was run in Tris/Glycine/SDS buffer (BioRad, USA) on a Mini-PROTEAN TGX Precast Gel (BioRad, USA) with Precision Plus Protein Kaleidoscope Prestained Protein Standards (Bio-Rad, USA). Proteins were transferred on a PVDF membrane using the Trans-Blot Turbo Transfer Pack (BioRad, USA). The membrane was blocked for 30 min in 5% milk in TBST buffer and incubated with mouse anti-HA antibody (#32-6700; Thermo Fisher Scientific, USA) in blocking solution (4 °C, overnight). Membranes were washed twice with 0.1% TBST buffer for 15 min and incubated with secondary rabbit anti-mouse HRP-conjugated antibody (ab6728 Abcam, Cambridge, UK) (1 h/RT). After washing, the membranes were visualized with a WesternBright Quantum (Advansta, San Jose, CA, USA) using G-Box (Syngene, Cambridge, UK). Anti-GAPDH primary antibody (ab9485 Abcam, UK) and secondary goat anti-rabbit (ab6721 Abcam, UK) were used as a loading control.

### 4.4. Cell Proliferation

To assess cell proliferation level after ZNF714 knockdown and overexpression, we used MTT colorimetric assay and real-time proliferation assay (IncuCyte^®^ S3, Sartorius, Göttingen, Germany). The MTT assay measured the number of viable cells as a ratio of cell metabolic activity relative to day one. We used an IncuCyte^®^ Instrument to monitor the real-time cell proliferation rate. Cells were seeded in an optimized amount on a 96-well culture plate and cultured in a humidified CO_2_ incubator inside an IncuCyte^®^ Instrument for three days. Pictures illustrating the level of confluency were taken every 3 h. The IncuCyte^®^ software (Incucyte-2021B software, Göttingen, Germany) allows comparison of the level of plate confluency at different time points and calculates the cell proliferation rate. The value of proliferation for each cell variant was normalized to the time point at which the control cells reached approximately 20% confluency. 

### 4.5. Cell Cycle and Apoptosis

We used flow cytometry-based assays to analyze cell cycle and apoptosis. The cell cycle was evaluated by staining all cell variants (WT, shLUC, shZNF714) with propidium iodide (PI) (Thermo Fisher Scientific, USA). Cells were harvested and washed in DPBS (Biowest, France). Obtained cell pellets were fixed in ice-cold 70% ethanol (POCH, Gliwice, Poland) for 30 min on ice with constant shaking. Afterward, samples were incubated with 5 μg of RNAse (EurX, Gdansk, Poland) and stained with 200 μL of PI (50 μg/mL) (30 min/37 °C). Cells were analyzed on FACSAria (BD Sciences, Franklin Lakes, NJ, USA). For apoptosis, we utilized a double staining protocol using PI in conjunction with Annexin V-APC (Thermo Fisher, USA). Harvested cells were suspended in 100 μL Annexin V Binding Buffer (Thermo Fisher, USA) with 5 μL of AnnV-APC and incubated for 15 min in the dark. After incubation, cells were washed and resuspended in 200 μL Annexin V Binding Buffer with 5 μL PI. After flow cytometry analysis, cells were divided into four categories: PI^−^/AnnV^−^ viable cells, PI^+^/AnnV^−^ necrotic cells, PI^+^/AnnV^+^ late apoptosis, and PI^−^/AnnV^+^ early apoptosis.

### 4.6. Migration and Invasion

Real-time migration and invasion assay for SKMES shZNF714 and ZNF714-OE cells were performed in the IncuCyte^®^ Instrument (Sartorius, USA). The procedure was performed according to the producer’s protocol. Briefly, 6 × 10^4^ cells were seeded on a plate coated with 100 µg/mL Matrigel™ (Thermo Fisher Scientific, USA) to obtain 100% confluency the next day. After overnight incubation in a humidified CO_2_ incubator, 3% mitomycin was added to stop cell proliferation. After 3 h, we used the WoundMaker device (Sartorius, USA) to create even wounds in every well. The cells were gently washed twice with PBS. Additionally, to assess cell invasion, the plate was incubated on ice for 5 min, and 50 μL of 2 mg/mL Matrigel™ was added. Cells were incubated for two days inside the IncuCyte Instrument. Migration and invasion were also tested by a Boyden chamber transmembrane assay with a chemoattractant (FBS, 10%) added to the bottom chamber of MultiScreen Filter Plates with 8 µm pores (Sigma Aldrich, USA). For the invasion assay, the membranes were coated with 2% Matrigel™. We seeded 25 × 10^3^ cells into the upper chamber. After overnight incubation, the cells remaining on the upper side of the membrane were scratched off with a cotton swab. The medium from the bottom chamber was further replaced with a medium containing 10% PrestoBlue reagent (Thermo Fisher Scientific, USA) to stain viable cells. Upon 3 h incubation (37 °C, 5% CO_2_), the fluorescence (Ex 560/Em 590 nm) was measured in a black-wall plate with a Victor plate reader (Perkin Elmer, Waltham, MA, USA).

### 4.7. Immunofluorescence Cell Staining

SKMES cells transduced with ZNF714-HA coding lentiviral vector (pEXPpSIN-TRE-GW-ZNF714-3xHA) were treated with 2 µg/mL doxycycline for 48 h to induce ZNF714 expression. Then, the cells were fixed with 4% paraformaldehyde (Agar Scientific Ltd., Stansted, UK) and permeabilized with ice-cold methanol, followed by blocking with 1% BSA in PBS for 15 min. The cells were further stained with anti-HA antibody (Thermo Fisher Scientific, USA) at a dilution of 1:100 and DyLight 650-labeled beta-actin monoclonal antibody (Thermo Fisher Scientific, USA) at 1:100 dilution overnight at 4 °C. Next, the cells were washed with 1% BSA in PBS and probed with Alexa Fluor^®^488-conjugated anti-rabbit secondary antibody (Jackson Immuno Research, West Grove, PA, USA) at a dilution of 1:200 for 1 h at room temperature. After washing with PBS, cells were immersed in a Fluoroshield mounting medium with DAPI (Sigma Aldrich, USA) to visualize cell nuclei. Pictures were taken with an Olympus Scanning Confocal Microscope FV1000 (Olympus, Shinjuku, Japan), connected to a laser diode at 405 nm and an argon laser. Image acquisition and analysis were performed with a 60× objective, a 1.4 NA oil immersion lens, and FLUOVIEW Viewer software (ver. 4.1, Olympus, Japan). 

### 4.8. ChIPseq

SKMES WT and ZNF714-OE cell variants were cultured with 2 µg/mL doxycycline for 48 h, trypsinized, washed with PBS, and counted. The chromatin immunoprecipitation was performed with the HighCell# ChIP Kit (Diagenode, Seraing, Belgium) according to the manufacturer’s protocol with minor modifications. Briefly, the lysates from 10 mln crosslinked (in 1% formaldehyde for 8 min) cells were sonicated with a Bioruptor device (Diagenode, Belgium) for 4 runs of 10 cycles (30 s on/30 s off). Before the immunoprecipitation, the lysates were purified on 50 µL pre-washed Pierce™ Protein A Agarose (Thermo Fisher Scientific, USA) for 2.5 h at 4 °C. Immunoprecipitation was performed using 2 µg µg rabbit anti-HA ChIP-grade antibody (#ab9110, Abcam, UK) during overnight incubation (4 °C, 40 rpm). For the DNA elution and decrosslinking, we used an iPURE kit (Diagenode, Belgium) according to the manufacturer’s instructions. DNA quantity was measured with the QuantiFluor dsDNA test (Promega, Madison, WI, USA). DNA quality assessment, sequencing, and subsequent bioinformatics analyses were outsourced to Novogene (Cambridge, UK). Paired-end (PE-150) sequencing of immunoprecipitated and input material was performed in duplicate on the Illumina NovaSeq platform (Illumina, San Diego, CA, USA). The Novogene bioinformatics pipeline included the following steps. Read quality control was performed with FastQC software (https://www.bioinformatics.babraham.ac.uk/projects/fastqc/, accessed on 5 September 2023). The reads were trimmed and mapped to the UCSC hg38 human genome with the BWA tool (v. 0.7.12), followed by a mapping quality check with MAPQ algorithms. MACS2 software (v. 2.1.0) was used for peak calling, which was further annotated with PeakAnnotator_Cpp (v.1.4).

### 4.9. RNAseq and Differential Gene Expression Analysis

RNA was isolated with a Quick-RNA Miniprep Kit (Zymo Research, Irvine, CA, USA) according to the manufacturer’s protocol. The quality and quantity of obtained samples were assessed with The RNA 6000 Nano assay on Bioanalyzer (Agilent Technologies, Santa Clara, CA, USA). Pair-end RNA sequencing was performed by Macrogen Europe on a NovaSeq 6000 Sequencing System (Illumina, USA). All samples for H2073 and SKMES cell lines (WT, shLUC, sh714) were sequenced in 3 biological replicates. Trimmed reads were mapped to the UCSC hg38 human reference genome with HISAT2. Based on the reference genome model, known genes and transcripts were assembled with StringTie. After assembly, the abundance of genes/transcripts was calculated in the read count and normalized value as FPKM (Fragments Per Kilobase of transcript per Million mapped reads). Expression profiles are represented as read counts and normalization values based on transcript length and depth of coverage. DEG (differentially expressed gene) analysis was performed on two different comparison pairs (sh714 vs. two control samples WT and shLUC separately) using edgeR. The results showed genes that satisfied the following conditions: |fc| >= 2 and exactTest raw *p*-value < 0.05. The fold change and the normalization were calculated using DESeq2. The transcriptomic data are available in the Gene Expression Omnibus (GEO) under the accession number GSE238088.

### 4.10. Gene Ontology, Cellular Component, and KEGG Pathway Analysis

Biological processes, cellular components, and KEGG analysis were performed with the DAVID (The Database for Annotation, Visualization and Integrated Discovery) database [78,79]. In the first step, we combined DEGs for shZNF714 obtained from two different comparisons, vs. WT and shLUC for each cell line separately. Further, we narrowed down the list only to those genes that were present in both datasets. We ended up with 807 DEGs for H2073 and 864 for the SKMES cell line. Ninety-seven genes were commonly deregulated in both tested cell lines, although some of them showed opposite patterns of down- or upregulation. Identified biological processes, cellular components, and KEGG pathways were considered significant at *p* < 0.05 and FDR < 0.25.

### 4.11. Methylation Microarray

Global methylation analysis was performed by Macrogen Europe with an Infinium Methylation EPIC BeadChIP Kit (Illumina, USA) on the Illumina iScan scanner. DNA from SKMES and H2073 (WT, shLUC, shZNF714) was isolated with a PureLink™ Genomic DNA Mini Kit (Thermo Fischer Scientific, USA) according to the manufacturer’s protocol and analyzed in biological triplicates. Each methylation data point is represented by fluorescent signals from the M (methylated) and U (unmethylated) alleles. Background intensity computed from a set of negative controls was subtracted from each analytical data point. The ratio of fluorescent signals was then calculated from the two alleles ß = (max(M, 0))/(|U| + |M| + 100). The ß-value (from 0 to 1) reflects the methylation level (from 0% to 100%, respectively) of each CpG site. Array data export processing and analysis were performed using Illumina GenomeStudio v2011.1 (Methylation Module v1.9.0) and R 4.2.2 (http://www.r-project.org, accessed on 16 January 2023). After raw data pre-processing and a quality check, statistical tests were performed (delta mean, independent *t*-test, odds ratio, and fold change). Differentially methylated CpGs (DM CpGs) were identified based on the delta mean, which was calculated as the difference between shZNF714 and control samples (WT or shLUC separately)(=mean(Avg_beta of Test group)-mean(Avg_beta of Control group)). Methylation changes were considered significant for delta mean > 0.2 at *p* < 0.05. The methylation data are freely available in the GEO repository under the accession number GSE238088.

### 4.12. RT-qPCR

RNA was isolated from 3 biological replicates of H2073 and SKMES cells (WT, shLUC, sh714) with a GeneMATRIX Universal RNA Purification Kit (EurX, Poland) according to the manufacturer’s protocol. Afterward, 1 ug of RNA was converted to cDNA with a smART First Strand cDNA Synthesis Kit (EurX, Poland). Gene expression analysis was performed using the LightCycler 480 SYBR Green Master Mix (Roche, Basel, Switzerland) or iTaq™ Universal SYBR^®^ Green Supermix (BioRad, USA) with 5 μM primer mix on a LightCycler 480 machine (Roche, Switzerland). ESD was used as an endogenous control in lung cancer cell lines and ACTB in breast cancer. Relative quantification was performed using the comparative Ct method (RQ = 2^−ΔΔCt^). We used the following primers: ESD: FW 5’-TGATCAAGGGAAAGATGACCA-3’, RV 5’-AACCCTCTTGCAATCGAAAA-3’; ACTB: FW 5’-CAGCCATGTACGTTGCTATCCAGG-3’, RV 5’-AGGTCCAGACGCAGGATGGCATG-3’; ZNF714: FW 5’-GCCCTGGAATATGAAGATATG-3’, RV5’-TTCTTAACTGTAAATTCTCATGT-3’; PCDH20: FW 5’-AGGAACCTGCCGCATCTGTT-3’, Rv 5’-GGGTAGTCCCTCGTTTAGGCT-3’; ARNT2: FW 5’-GATTGGAACGAGCCACACCT-3’, Rv 5’-TGTCCACTTTCAGCGAACCC-3’; COL8A1: FW 5’-GAGTATCCACACCTACCCCA-3’, Rv 5’-CGTAAACTGGCTAATGGTATT-3’; EFEMP2: FW 5’-ACAGCTACACGGAATGCACA-3’, Rv 5’-CCCGTAGTGGTTGATGCACT-3’; ITGA7: FW 5’-TGGTTGGGAGTCAGTGTTCG-3’, Rv 5’-CTGCCTTGCCTCATATCGGT-3’; IGFBP3: FW 5’-GCGGGCTCTGCGTCAAC-3’, Rv 5’-TCCTCCGACTCACTAGCATTTC-3’; TIMP3: FW 5’-GCGCAAGGGGCTGAACTATC-3’, Rv 5’-TCGGTCCAGAGACACTCGTT-3’; STRA6: FW 5’-CCATCTGTGGGCTCTGGAAG-3’, Rv 5’-TCGAAGGTTGGTCCTGTGTG-3’.

### 4.13. Methylation-Sensitive High Resolution Melting (MS-HRM)

To validate the methylation microarray results, we utilized the MS-HRM method. DNA from 3 biological replicates of H2073 and SKMES cells (WT, shLUC, sh714) was isolated with the Cell Culture DNA Purification Kit (EurX, Poland) according to the manufacturer’s protocol. In the next step, 500 ng–1800 ng of DNA was subjected to bisulfite conversion with the EZ DNA Methylation-Gold Kit (Zymo Research, USA). MS-HRM reaction was performed on a LightCycler 480 machine (Roche, Switzerland) using the HRM PCR Master Mix (EurX, Poland). The results were calculated using the Gene Scanning Module of LightCycler 480 Software (ver. 1.5.1.62) and presented as normalized and shifted melting curves. We used the following primers: PCDH20: FW 5′-AATAAATAAGTTTGTATTTGTATATGGATG-3′; Rv 5′-TTTCTCTATACCAACACTACTAAATCC-3′; STRA6: FW 5′-ATTTTAGGAGGAGTAGGAGGTAG-3′, Rv 5′-ACAAAAAAATAACTCACCCCCACA-3′; ARNT2: FW 5′-TGTTAAGTGGGTAGTATTGG-3′, Rv 5′-CCCATTTAAATTCAAAAAAAAAC-3′; TIMP3: FW 5′-AGATATTTAGTGGTTTAGGTGGG-3′, Rv 5′-TTCAAATCCTTATAAAAAATAATACC-3′.

## 5. Conclusions

In summary, we showed that ZNF714 is overexpressed in multiple tumors, likely due to regional amplification, promoter hypomethylation, and NFYB signaling. In the in vitro studies, ZNF714 showed a positive influence on cell proliferation, migration, and invasion, whereas transcriptomic profiling pointed towards the deregulation of the genes involved in those processes. Moreover, we showed that ZNF714 negatively regulates the expression of a number of known TSGs via promoter methylation, although this effect is indirect. According to our knowledge, this is the first report exhibiting an oncogenic role of ZNF714. Nevertheless, further studies using animal models and other tumor types are needed to better understand the role of ZNF714 in a cancer setting.

## Figures and Tables

**Table 1:** Differential gene expression and DNA methylation of analyzed target genes based on RNA-seq and methylation microarray, respectively.

	Expression FC shZNF714/WT	Expression FC shZNF714/shLUC	Probes ID	CpG Methylation Difference shZNF714-WT	CpG Methylation Difference shZNF714-shLUC
PCDH20	2.34	3.30	cg02246190	−0.22	−0.24
			cg05034702	−0.56	−0.48
			cg15182360	−0.35	−0.30
			cg00245538	−0.34	−0.29
			cg17903776	−0.24	−0.28
			cg12721730	NA	−0.24
			cg15839219	−0.26	−0.22
STRA6	8.15	10.70	cg13888509	−0.54	−0.50
			cg18640660	−0.39	−0.35
			cg22672067	−0.28	−0.26
			cg26774156	−0.63	−0.59
			cg00075967	−0.47	−0.48
			cg15162876	−0.32	−0.32
			cg14275207	−0.50	−0.44
ARNT2	5.44	2.78	cg05201477	−0.28	−0.28
			cg20775959	−0.29	−0.25
TIMP3	5.82	3.19	cg14456116	−0.46	−0.42
			cg15004938	−0.41	−0.38

## Data Availability

The transcriptomic RNAseq data are available in the Gene Expression Omnibus (GEO) under the accession number GSE238088. The methylation data are freely available in the GEO repository under the accession number GSE238088.

## Supplementary Materials

The following supporting information can be downloaded at: https://www.mdpi.com/article/10.3390/ijms242115530/s1.
