# Supplementary material for: ZNF714 Supports Pro-Oncogenic Features in Lung Cancer Cells
Source: Int J Mol Sci. 2023 Oct 24;24(21):15530. doi: 10.3390/ijms242115530 (PMC10649060; doi:10.3390/ijms242115530)
Supplement: Supplementary file 1 [file ijms-24-15530-s001.zip › Supplemental figure 1.pptx]

## Slide 1
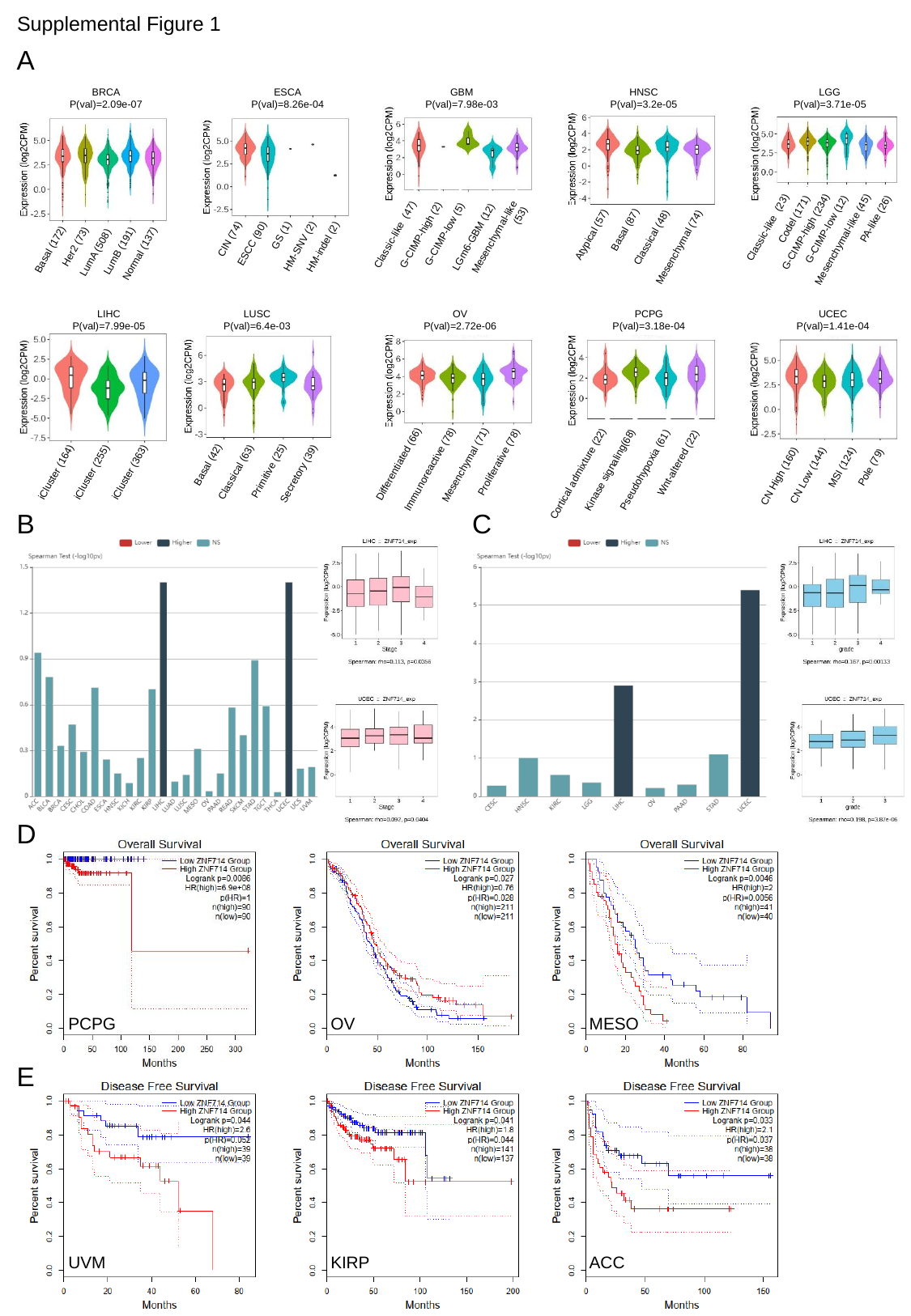

Supplemental Figure 1
A
BRCA
P(val)=2.09e-07
ESCA
P(val)=8.26e-04
GBM
P(val)=7.98e-03
HNSC
P(val)=3.2e-05
LGG
P(val)=3.71e-05
PA-like (26)
Codel (171)
G-CIMP-low (12)
G-CIMP-low (5)
G-CIMP-high (234)
Classic-like (23)
Classical (48)
Mesenchymal-like (53)
LGm6-GBM (12)
G-CIMP-high (2)
Classic-like (47)
Atypical (57)
Basal (87)
Mesenchymal-like (45)
Mesenchymal (74)
Her2 (73)
CIN (74)
ESCC (90)
GS (1)
HM-SNV (2)
HM-indel (2)
LumA (508)
Normal (137)
Basal (172)
LumB (191)
LIHC
P(val)=7.99e-05
LUSC
P(val)=6.4e-03
OV
P(val)=2.72e-06
PCPG
P(val)=3.18e-04
UCEC
P(val)=1.41e-04
Proliferative (78)
Wnt-altered (22)
Differentiated (66)
Mesenchymal (71)
iCluster (164)
iCluster (363)
iCluster (255)
Pseudohypoxia (61)
Immunoreactive (78)
Classical (63)
Kinase signaling(68)
Primitive (25)
Basal (42)
Cortical admixture (22)
Secretory (39)
Pole (79)
CN High (160)
MSI (124)
CN Low (144)
B
C
D
PCPG
OV
MESO
UVM
KIRP
ACC
E

## Slide 2
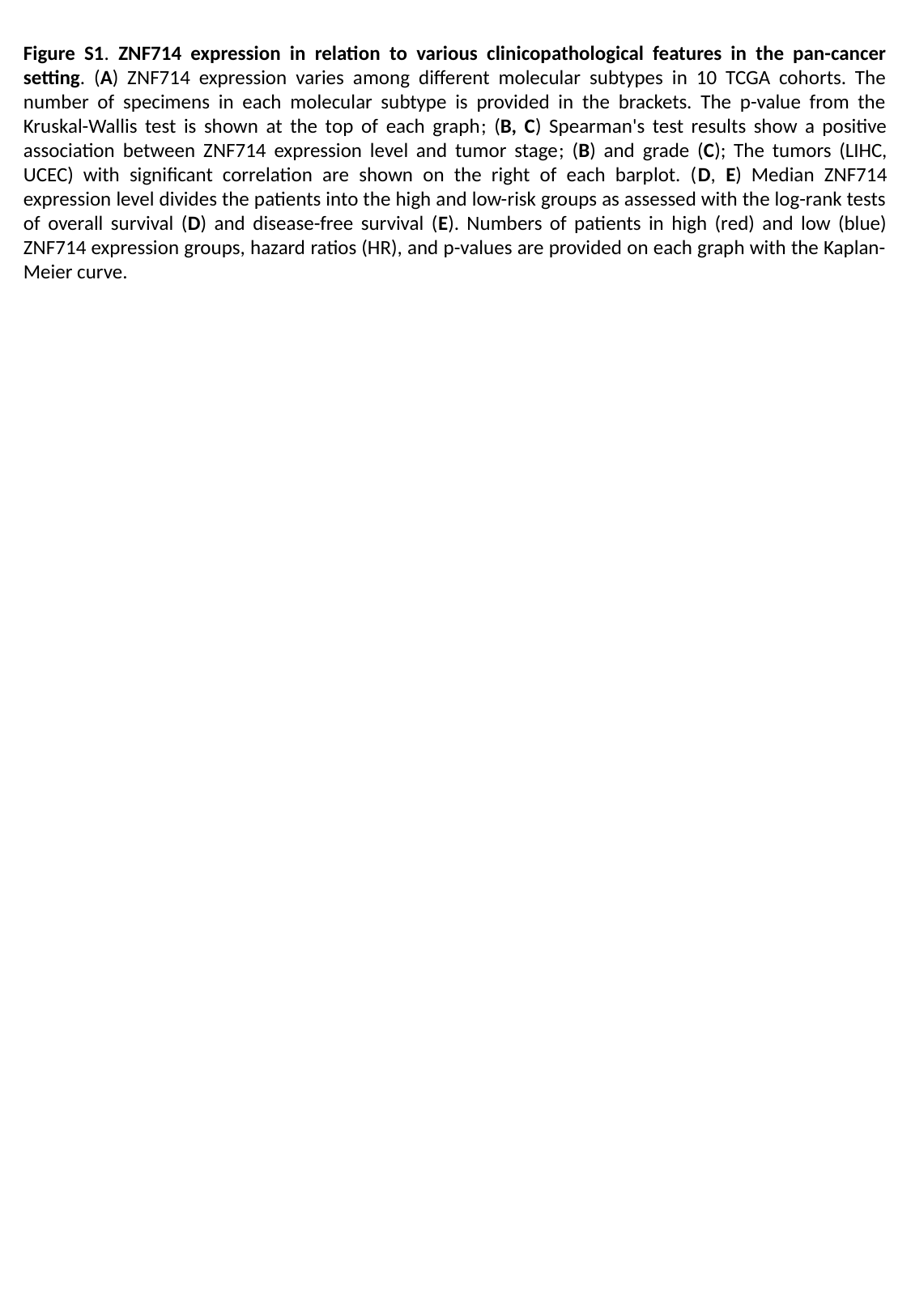

Figure S1. ZNF714 expression in relation to various clinicopathological features in the pan-cancer setting. (A) ZNF714 expression varies among different molecular subtypes in 10 TCGA cohorts. The number of specimens in each molecular subtype is provided in the brackets. The p-value from the Kruskal-Wallis test is shown at the top of each graph; (B, C) Spearman's test results show a positive association between ZNF714 expression level and tumor stage; (B) and grade (C); The tumors (LIHC, UCEC) with significant correlation are shown on the right of each barplot. (D, E) Median ZNF714 expression level divides the patients into the high and low-risk groups as assessed with the log-rank tests of overall survival (D) and disease-free survival (E). Numbers of patients in high (red) and low (blue) ZNF714 expression groups, hazard ratios (HR), and p-values are provided on each graph with the Kaplan-Meier curve.
