# Supplementary material for: ZNF714 Supports Pro-Oncogenic Features in Lung Cancer Cells
Source: Int J Mol Sci. 2023 Oct 24;24(21):15530. doi: 10.3390/ijms242115530 (PMC10649060; doi:10.3390/ijms242115530)
Supplement: Supplementary file 1 [file ijms-24-15530-s001.zip › Supplemental figure 2.pptx]

## Slide 1
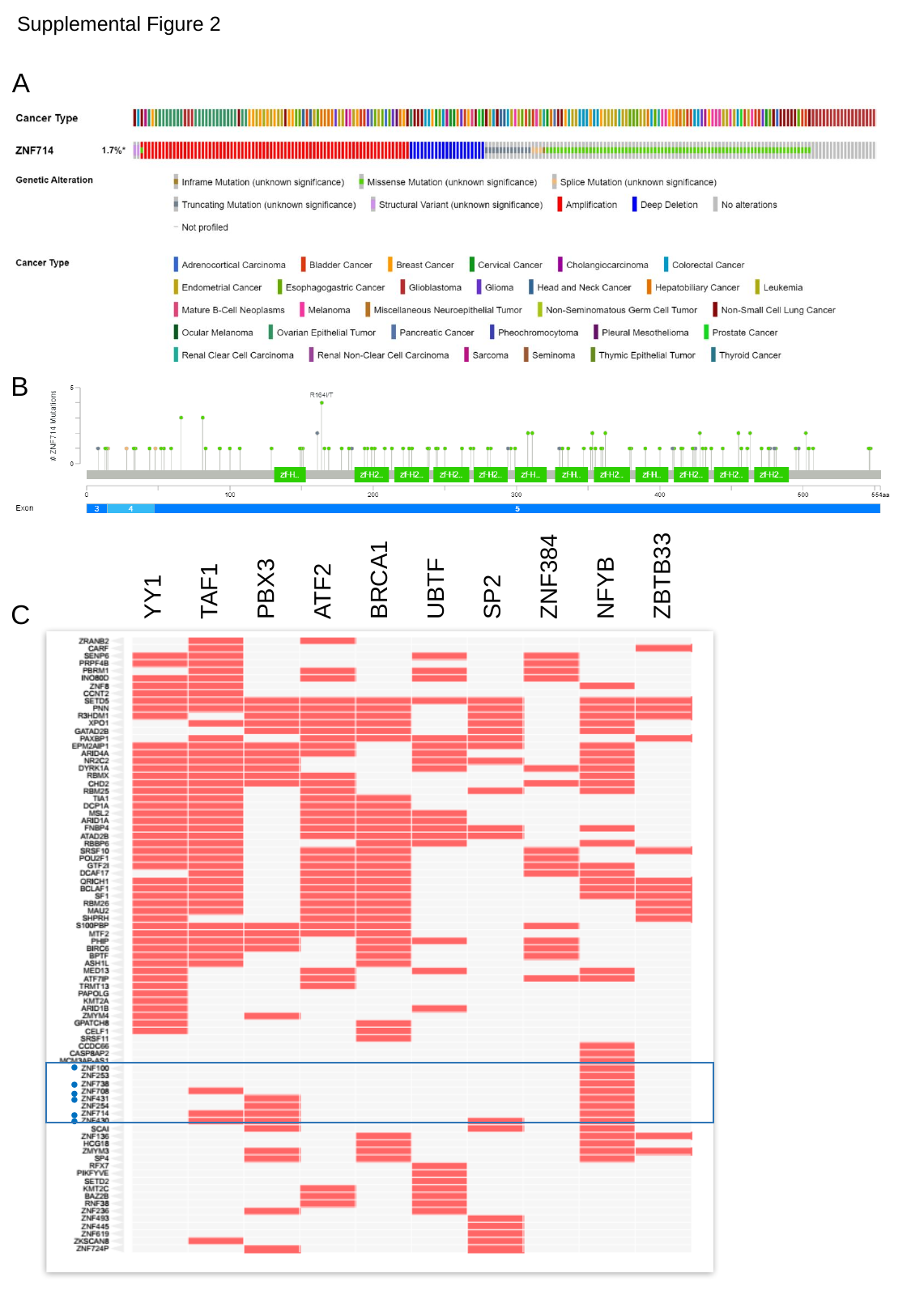

Supplemental Figure 2
A
B
YY1
TAF1
PBX3
ATF2
BRCA1
UBTF
SP2
ZNF384
NFYB
ZBTB33
C

## Slide 2
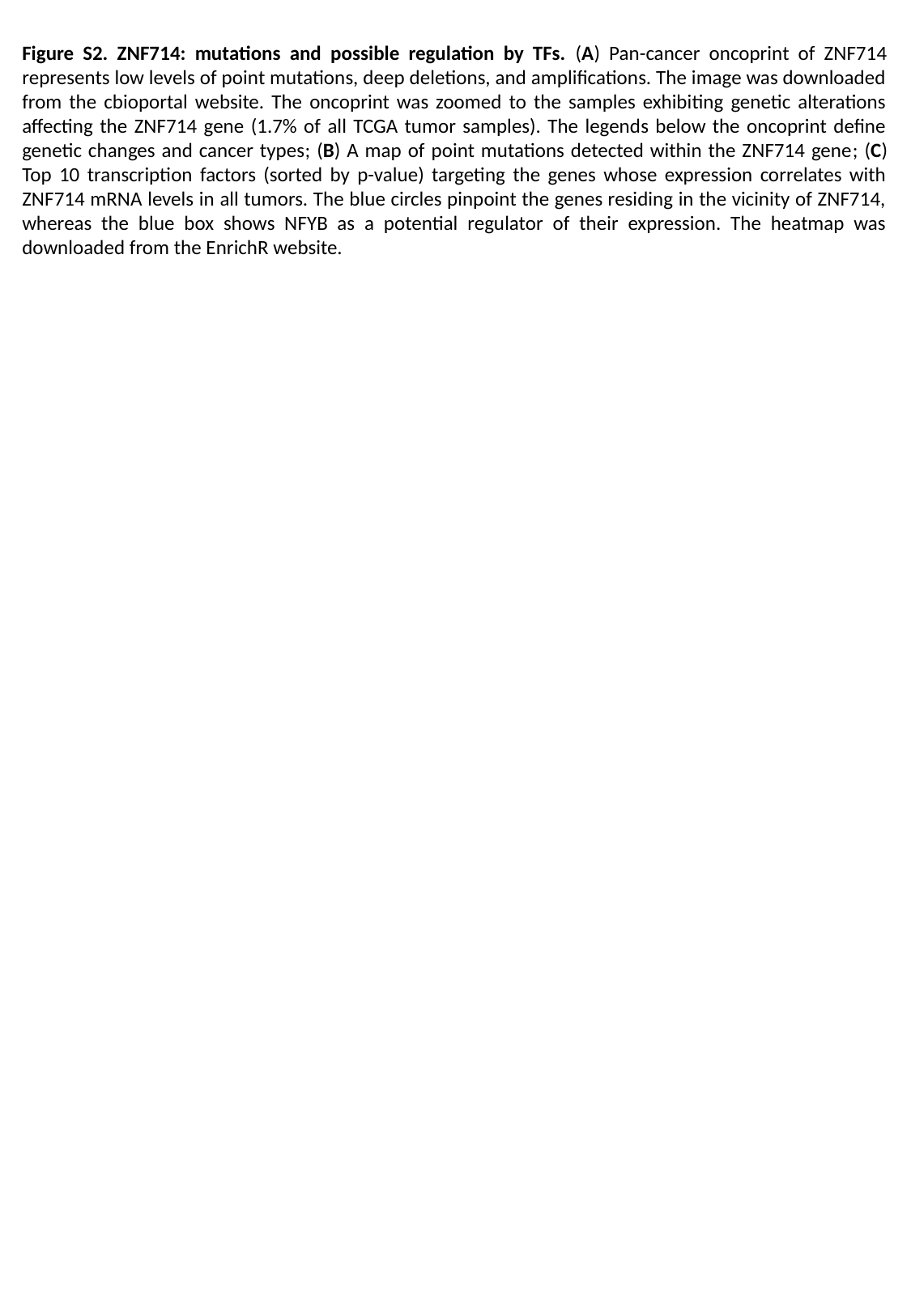

Figure S2. ZNF714: mutations and possible regulation by TFs. (A) Pan-cancer oncoprint of ZNF714 represents low levels of point mutations, deep deletions, and amplifications. The image was downloaded from the cbioportal website. The oncoprint was zoomed to the samples exhibiting genetic alterations affecting the ZNF714 gene (1.7% of all TCGA tumor samples). The legends below the oncoprint define genetic changes and cancer types; (B) A map of point mutations detected within the ZNF714 gene; (C) Top 10 transcription factors (sorted by p-value) targeting the genes whose expression correlates with ZNF714 mRNA levels in all tumors. The blue circles pinpoint the genes residing in the vicinity of ZNF714, whereas the blue box shows NFYB as a potential regulator of their expression. The heatmap was downloaded from the EnrichR website.
