# Supplementary material for: ZNF714 Supports Pro-Oncogenic Features in Lung Cancer Cells
Source: Int J Mol Sci. 2023 Oct 24;24(21):15530. doi: 10.3390/ijms242115530 (PMC10649060; doi:10.3390/ijms242115530)
Supplement: Supplementary file 1 [file ijms-24-15530-s001.zip › Supplemental figure 3.pptx]

## Slide 1
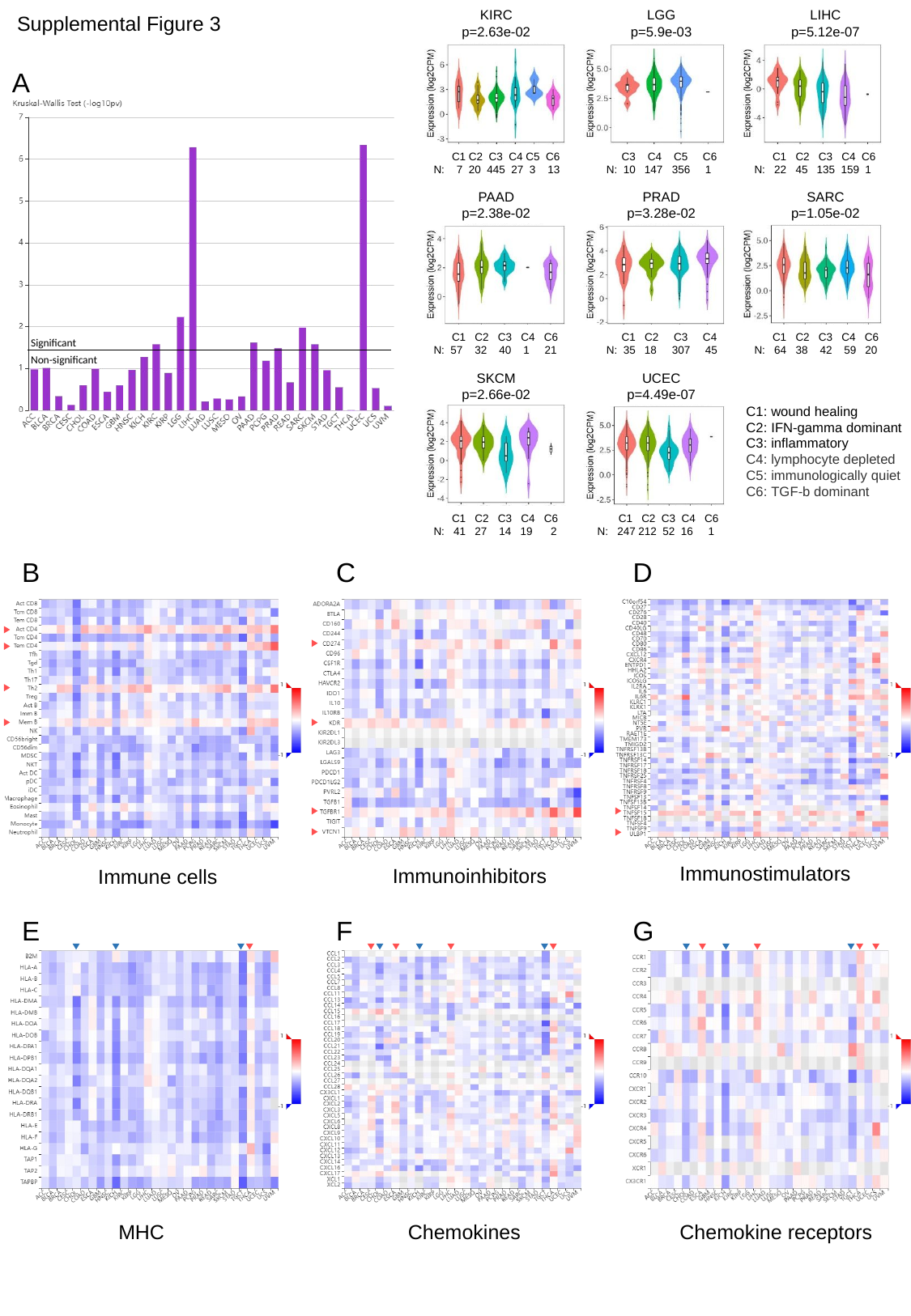

KIRC
p=2.63e-02
LGG
p=5.9e-03
LIHC
p=5.12e-07
Supplemental Figure 3
A
 C1 C2 C3 C4 C5 C6
 N: 7 20 445 27 3 13
 C3 C4 C5 C6
 N: 10 147 356 1
 C1 C2 C3 C4 C6
 N: 22 45 135 159 1
PAAD
p=2.38e-02
PRAD
p=3.28e-02
SARC
p=1.05e-02
 C1 C2 C3 C4 C6
 N: 57 32 40 1 21
 C1 C2 C3 C4
 N: 35 18 307 45
 C1 C2 C3 C4 C6
 N: 64 38 42 59 20
Significant
Non-significant
SKCM
p=2.66e-02
UCEC
p=4.49e-07
C1: wound healing
C2: IFN-gamma dominant
C3: inflammatory
C4: lymphocyte depleted
C5: immunologically quiet
C6: TGF-b dominant
 C1 C2 C3 C4 C6
 N: 41 27 14 19 2
 C1 C2 C3 C4 C6
N: 247 212 52 16 1
B
C
D
Immunostimulators
Immunoinhibitors
Immune cells
E
F
G
MHC
Chemokines
Chemokine receptors

## Slide 2
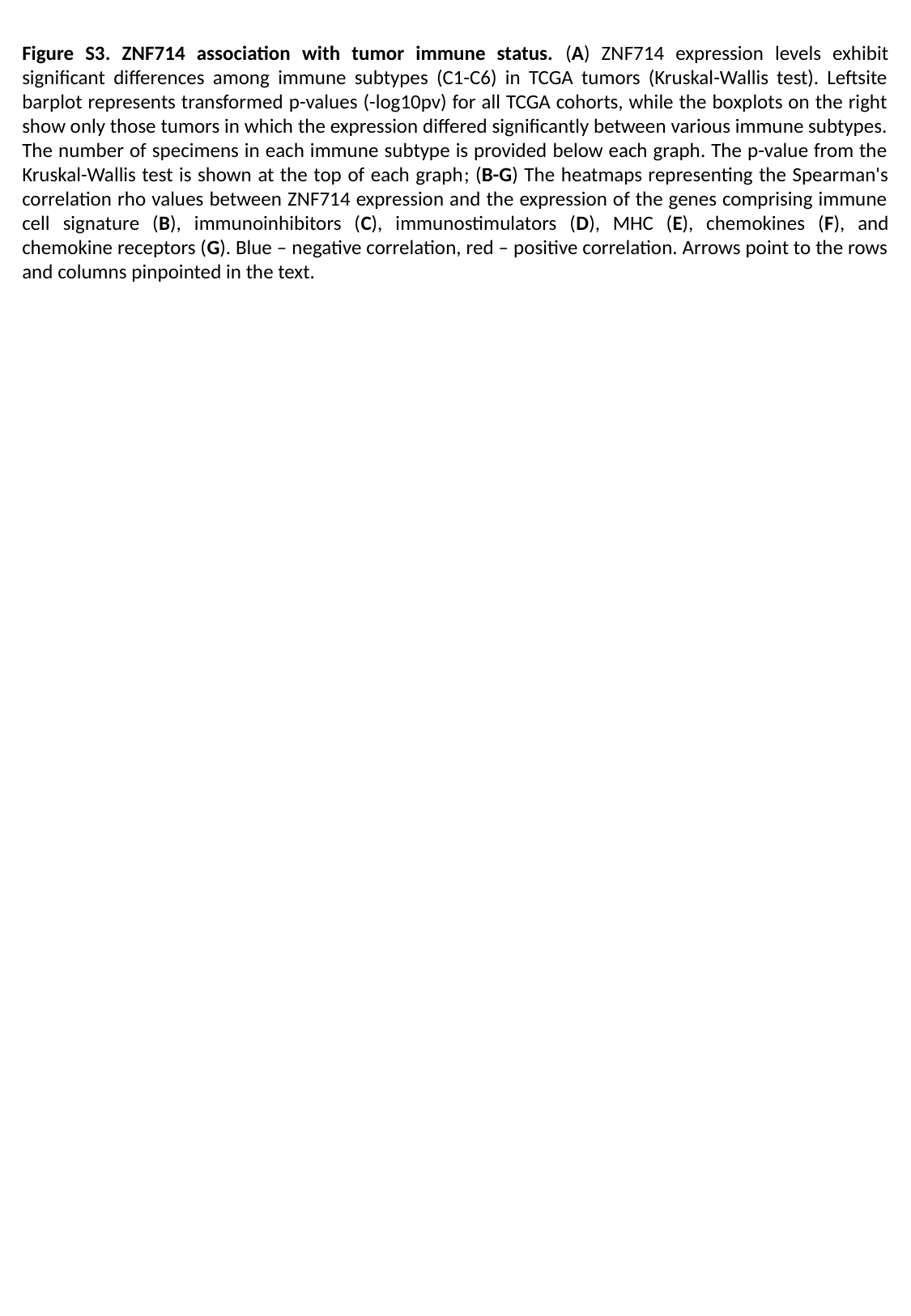

Figure S3. ZNF714 association with tumor immune status. (A) ZNF714 expression levels exhibit significant differences among immune subtypes (C1-C6) in TCGA tumors (Kruskal-Wallis test). Leftsite barplot represents transformed p-values (-log10pv) for all TCGA cohorts, while the boxplots on the right show only those tumors in which the expression differed significantly between various immune subtypes. The number of specimens in each immune subtype is provided below each graph. The p-value from the Kruskal-Wallis test is shown at the top of each graph; (B-G) The heatmaps representing the Spearman's correlation rho values between ZNF714 expression and the expression of the genes comprising immune cell signature (B), immunoinhibitors (C), immunostimulators (D), MHC (E), chemokines (F), and chemokine receptors (G). Blue – negative correlation, red – positive correlation. Arrows point to the rows and columns pinpointed in the text.
