# Supplementary material for: ZNF714 Supports Pro-Oncogenic Features in Lung Cancer Cells
Source: Int J Mol Sci. 2023 Oct 24;24(21):15530. doi: 10.3390/ijms242115530 (PMC10649060; doi:10.3390/ijms242115530)
Supplement: Supplementary file 1 [file ijms-24-15530-s001.zip › Supplemental figure 4.pptx]

## Slide 1
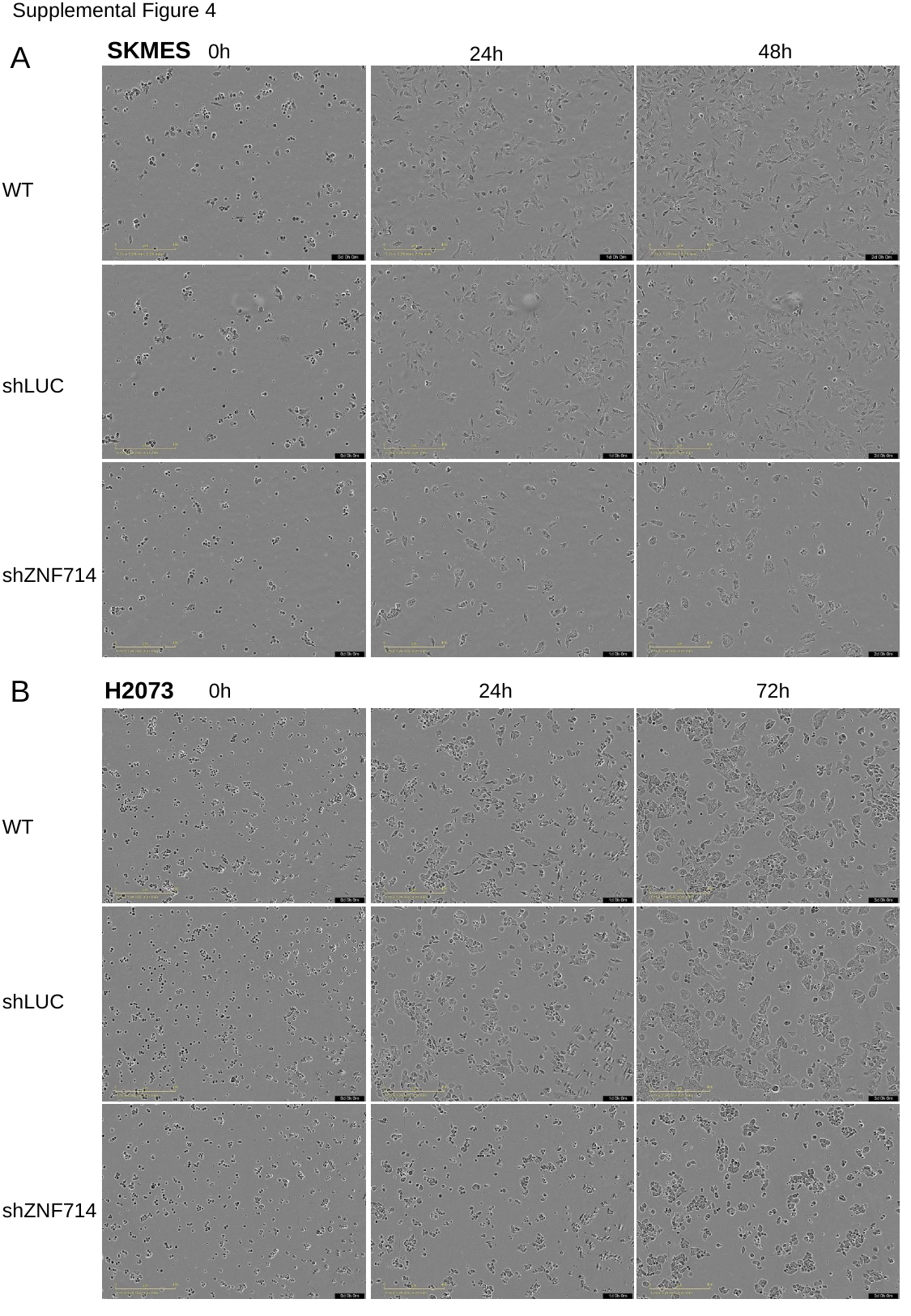

Supplemental Figure 4
SKMES
A
0h
48h
24h
WT
shLUC
shZNF714
B
H2073
0h
24h
72h
WT
shLUC
shZNF714

## Slide 2
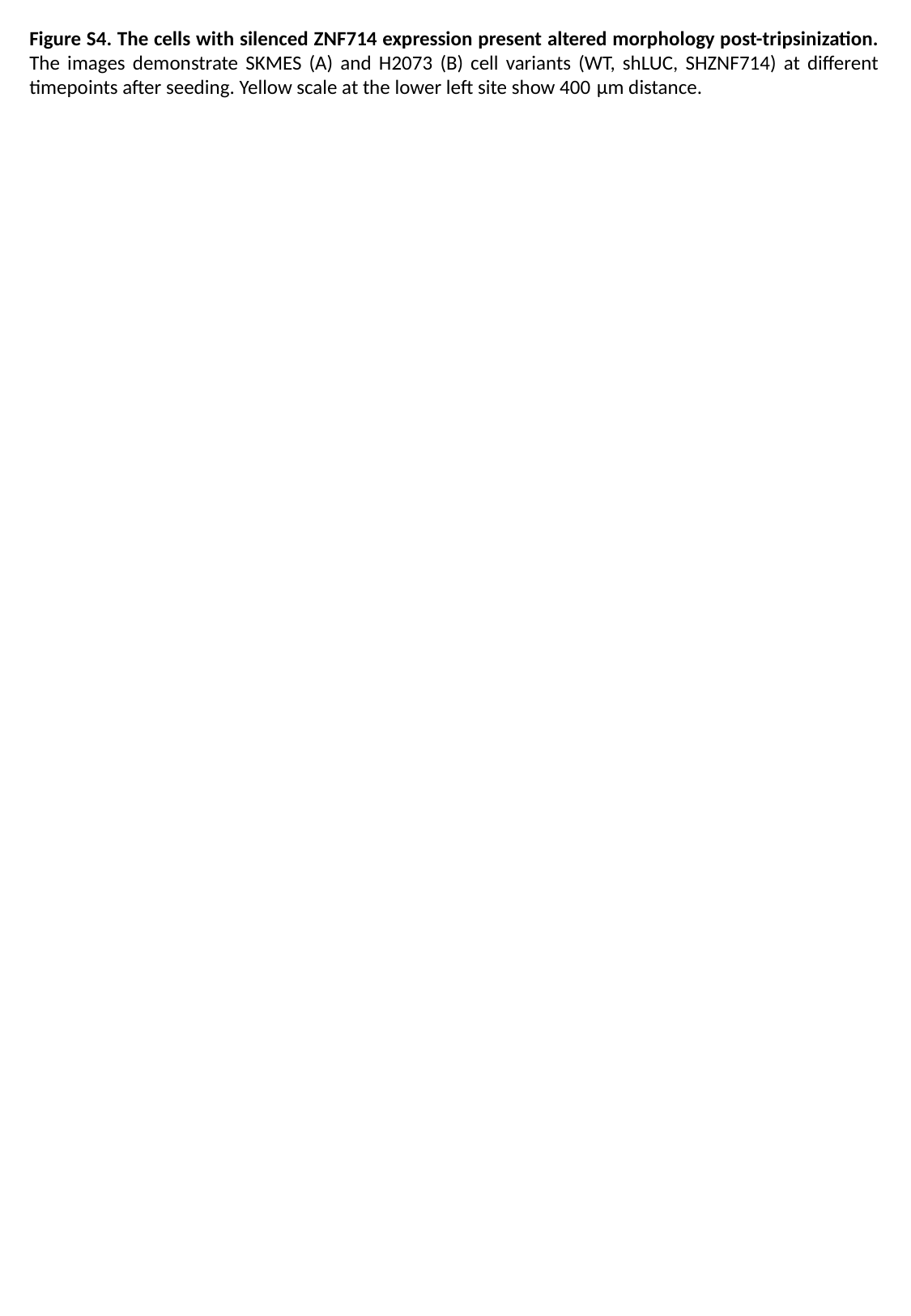

Figure S4. The cells with silenced ZNF714 expression present altered morphology post-tripsinization. The images demonstrate SKMES (A) and H2073 (B) cell variants (WT, shLUC, SHZNF714) at different timepoints after seeding. Yellow scale at the lower left site show 400 μm distance.
