# Supplementary material for: ZNF714 Supports Pro-Oncogenic Features in Lung Cancer Cells
Source: Int J Mol Sci. 2023 Oct 24;24(21):15530. doi: 10.3390/ijms242115530 (PMC10649060; doi:10.3390/ijms242115530)
Supplement: Supplementary file 1 [file ijms-24-15530-s001.zip › Supplemental figure 5.pptx]

## Slide 1
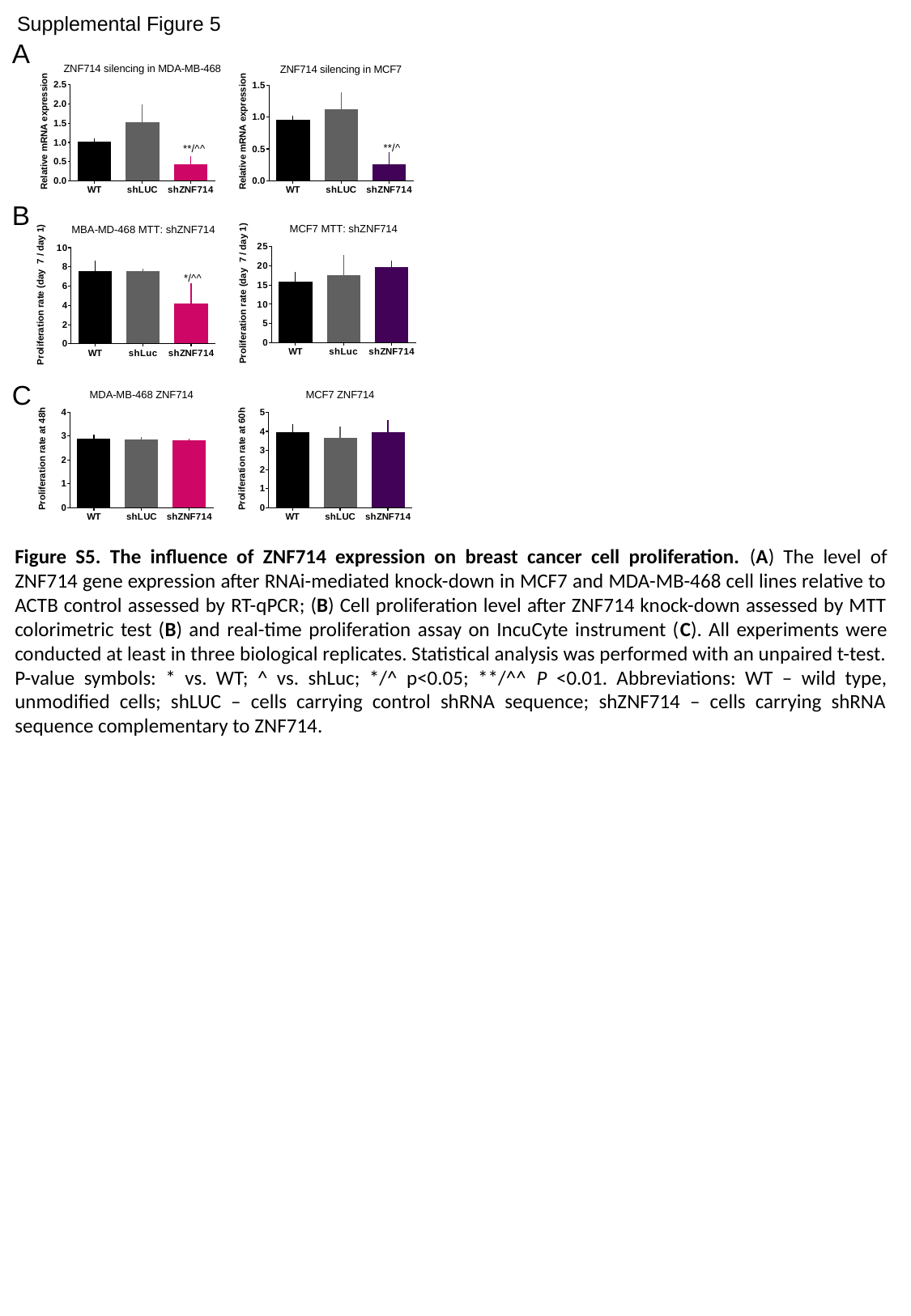

Supplemental Figure 5
A
B
C
Figure S5. The influence of ZNF714 expression on breast cancer cell proliferation. (A) The level of ZNF714 gene expression after RNAi-mediated knock-down in MCF7 and MDA-MB-468 cell lines relative to ACTB control assessed by RT-qPCR; (B) Cell proliferation level after ZNF714 knock-down assessed by MTT colorimetric test (B) and real-time proliferation assay on IncuCyte instrument (C). All experiments were conducted at least in three biological replicates. Statistical analysis was performed with an unpaired t-test. P-value symbols: * vs. WT; ^ vs. shLuc; */^ p<0.05; **/^^ P <0.01. Abbreviations: WT – wild type, unmodified cells; shLUC – cells carrying control shRNA sequence; shZNF714 – cells carrying shRNA sequence complementary to ZNF714.
