# Supplementary material for: ZNF714 Supports Pro-Oncogenic Features in Lung Cancer Cells
Source: Int J Mol Sci. 2023 Oct 24;24(21):15530. doi: 10.3390/ijms242115530 (PMC10649060; doi:10.3390/ijms242115530)
Supplement: Supplementary file 1 [file ijms-24-15530-s001.zip › Supplemental figure 6.pptx]

## Slide 1
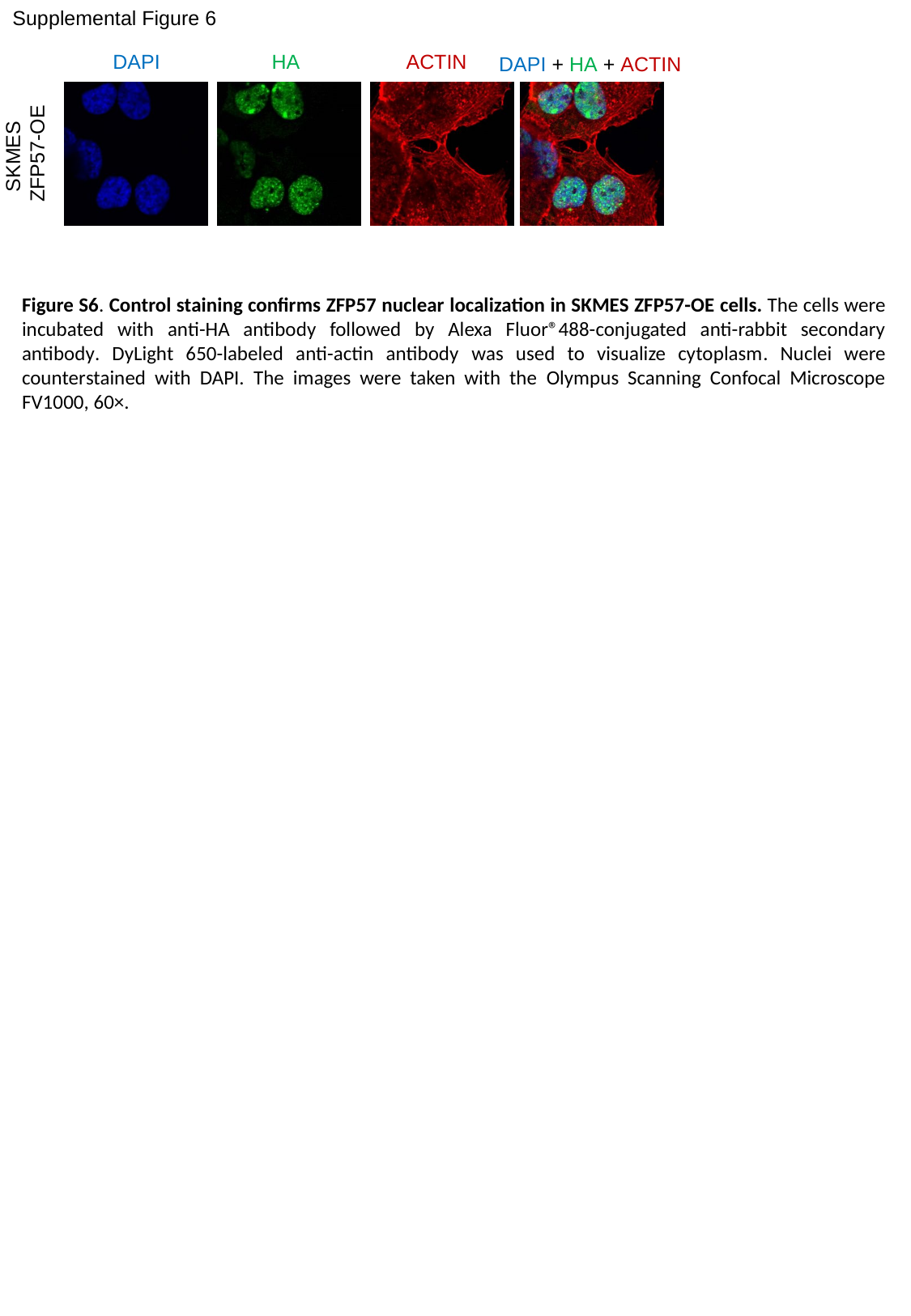

Supplemental Figure 6
DAPI
HA
ACTIN
DAPI + HA/+ ACTIN
SKMES
ZFP57-OE
Figure S6. Control staining confirms ZFP57 nuclear localization in SKMES ZFP57-OE cells. The cells were incubated with anti-HA antibody followed by Alexa Fluor®488-conjugated anti-rabbit secondary antibody. DyLight 650-labeled anti-actin antibody was used to visualize cytoplasm. Nuclei were counterstained with DAPI. The images were taken with the Olympus Scanning Confocal Microscope FV1000, 60×.
