# Supplementary material for: ZNF714 Supports Pro-Oncogenic Features in Lung Cancer Cells
Source: Int J Mol Sci. 2023 Oct 24;24(21):15530. doi: 10.3390/ijms242115530 (PMC10649060; doi:10.3390/ijms242115530)
Supplement: Supplementary file 1 [file ijms-24-15530-s001.zip › Supplemental figure 7.pptx]

## Slide 1
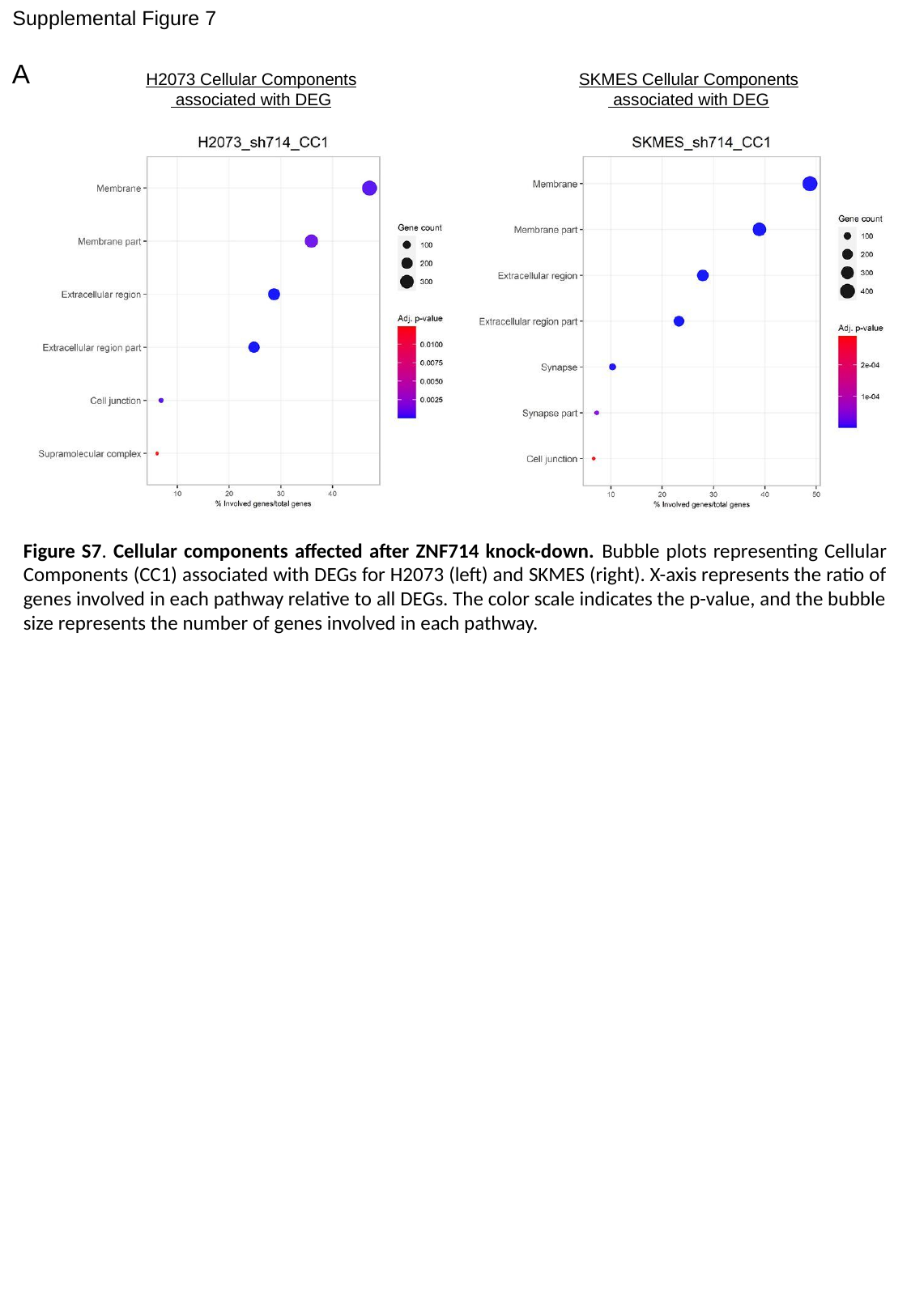

Supplemental Figure 7
A
H2073 Cellular Components
 associated with DEG
SKMES Cellular Components
 associated with DEG
Figure S7. Cellular components affected after ZNF714 knock-down. Bubble plots representing Cellular Components (CC1) associated with DEGs for H2073 (left) and SKMES (right). X-axis represents the ratio of genes involved in each pathway relative to all DEGs. The color scale indicates the p-value, and the bubble size represents the number of genes involved in each pathway.
